# Analytics and visualization tools to characterize single-cell stochasticity using bacterial single-cell movie cytometry data

**DOI:** 10.1186/s12859-021-04409-9

**Published:** 2021-10-29

**Authors:** Athanasios D. Balomenos, Victoria Stefanou, Elias S. Manolakos

**Affiliations:** 1 Department of Informatics and Telecommunications, National and Kapodistrian University of Athens, Ilissia, Greece; 2 Bouvé College of Health Sciences, Northeastern University, Boston, USA

**Keywords:** Time-lapse microscopy, Live-cell imaging, Lineage trees, Generation trees, Cell cytometry, Single-cell analytics, Visualization, Stochasticity modeling, Bacterial cell community dynamics

## Abstract

**Background:**

Time-lapse microscopy live-cell imaging is essential for studying the evolution of bacterial communities at single-cell resolution. It allows capturing detailed information about the morphology, gene expression, and spatial characteristics of individual cells at every time instance of the imaging experiment. The image analysis of bacterial "single-cell movies" (videos) generates big data in the form of multidimensional time series of measured bacterial attributes. If properly analyzed, these datasets can help us decipher the bacterial communities' growth dynamics and identify the sources and potential functional role of intra- and inter-subpopulation heterogeneity. Recent research has highlighted the importance of investigating the role of biological "noise" in gene regulation, cell growth, cell division, etc. Single-cell analytics of complex single-cell movie datasets, capturing the interaction of multiple micro-colonies with thousands of cells, can shed light on essential phenomena for human health, such as the competition of pathogens and benign microbiome cells, the emergence of dormant cells (“persisters”), the formation of biofilms under different stress conditions, etc. However, highly accurate and automated bacterial bioimage analysis and single-cell analytics methods remain elusive, even though they are required before we can routinely exploit the plethora of data that single-cell movies generate.

**Results:**

We present visualization and single-cell analytics using R (ViSCAR), a set of methods and corresponding functions, to visually explore and correlate single-cell attributes generated from the image processing of complex bacterial single-cell movies. They can be used to model and visualize the spatiotemporal evolution of attributes at different levels of the microbial community organization (i.e., cell population, colony, generation, etc.), to discover possible epigenetic information transfer across cell generations, infer mathematical and statistical models describing various stochastic phenomena (e.g., cell growth, cell division), and even identify and auto-correct errors introduced unavoidably during the bioimage analysis of a dense movie with thousands of overcrowded cells in the microscope's field of view.

**Conclusions:**

ViSCAR empowers researchers to capture and characterize the stochasticity, uncover the mechanisms leading to cellular phenotypes of interest, and decipher a large heterogeneous microbial communities' dynamic behavior. ViSCAR source code is available from GitLab at https://gitlab.com/ManolakosLab/viscar.

**Supplementary Information:**

The online version contains supplementary material available at 10.1186/s12859-021-04409-9.

## Background

Each cell in a microbial community is unique. While much of this diversity is due to genetic differences, even isogenic cells exposed to the same environmental conditions exhibit remarkable variability in their phenotypic characteristics. This heterogeneity is linked to the inherently probabilistic nature of molecular processes not involving changes in the genome. For example, the production of a specific protein among clonal cells can differ due to stochastic fluctuations (biological “noise”) during transcription and translation, leading to differences in protein levels in time and space [1].

It is well established that cell-to-cell variability in isogenic microbial cultures provides the flexibility cells need to adapt efficiently to changing environments [2, 3]. For example, “persister” cells switch between phenotypic states of different growth rates to achieve tolerance to antibiotics [4]. Moreover, pathogen biofilms [5] (Salmonella, Chlamydia, Escherichia coli, Staphylococcus, and Streptococcus) benefit from the creation of variant subpopulations of dormant persisters [4, 6, 7] that can survive an extended period of exposure to drugs. When most of the cell population is demised, the surviving persister cells transit out of their dormant state, causing the infection to re-emerge after the drug treatment is removed.

Studying the dynamical behavior of microbial communities has been a significant challenge in the post-genomic era to uncover the interactions and mechanisms leading to cell phenotypes of interest for human health [8–12]. Systems microbiology research has contributed models to characterize microbial populations’ growth. However, the existing heterogeneity at the single-cell level is masked in conventional studies. Therefore, more recent studies emphasize monitoring single-cell microbial kinetics and developing stochastic dynamical models that can provide more realistic predictions on cell community behavior under different conditions [1, 5–8, 11, 13–15].

With the realization that bacteria display phenotypic variability and exhibit complex subcellular organization critical for cellular function, optical microscopy has re-emerged as a primary tool for studying living cells [16, 17]. State-of-the-art microscopes and sensitive cameras paired with powerful genetically encoded fluorescent probes [18, 19] allow for high-resolution real-time observation of biological processes in vivo [20–23]. Time-lapse microscopy allows us today to monitor bacterial communities at a single-cell resolution over a period of time using phase-contrast and/or fluorescence technologies [24–26]. The generated videos, which from now on we will call “single-cell movies,” capture how the single-cells’ biophysical and/or gene expression properties evolve in space and time. Exploration of the multidimensional datasets the image analysis of single-cell movie videos generates is necessary to shed light on how the stochasticity (biological “noise”) affects essential phenomena in biology, such as cell proliferation, division, bacteria-host interaction, etc. [24–28].

Besides, the live-imaging of microbial consortia has the potential to uncover the stochastic processes involved in critical phenomena for human health, such as the competition between pathogens and benign microbiome cells [29], dormant cells (persisters) emergence under different stress conditions [5–7], etc. However, accurate, automated, and integrated image analysis and single-cell data analytics pipelines are lacking. They are required before we can harness the large body of single-cell movies created at different labs to generate realistic digital replicas of dynamic cell communities, also known as “digital twins” [30, 31]. If faithful to the physical world's relevant aspects, such virtual representations can empower systems and synthetic biology research, biotechnology advancements, and accelerate drug discovery and therapeutic strategies development. Active microscopy and advanced computation of cytometry data produced in real-time, i.e., as we live-image interacting bacterial micro-colonies (microbial consortia) [29, 32], will help us uncover the “logic” of critical biological phenomena currently escaping our understanding.

Prerequisites for the useful analysis of single-cell movies are the accurate cell segmentation and cell tracking. Segmentation identifies the individual cell regions, i.e., assigns pixels in an image frame to individual cells. Tracking, on the other hand, associates corresponding cell regions from one frame to the next. Without completing these two essential image analysis tasks successfully, it is impossible to characterize the cellular morphology and/or fluorescence levels and, therefore, convert the data emanating from live-cell imaging experiments into a suitable digital representation of living communities’ properties at single-cell resolution. It is the success of this transformation that can allow us to eventually retire the single-cell movies themselves and use their digital replicas to feed powerful analytics pipelines for downstream analysis.

Single-cell movies’ analysis initially relied on a visual inspection and laborious manual annotation, being equally time-consuming or even more tedious than the sample preparation and imaging tasks themselves. It is undeniable that automated bioimage analysis pipelines [33] that routinely accomplish accurate cell segmentation, tracking, and lineage trees construction are needed to exploit the abundance of single-cell movies and extract consistent and reliable information from them without requiring significant human effort. Along these lines, several computational methods and tools have been developed for analyzing bacterial single-cell movies. These include, among others, the TLM-Tracker [34], CellTracer [35], MicrobeTracker [36] and its successor Oufti [37], Schnitzcells [38], and more recently MicrobeJ [39], SuperSegger [40], BacStalk [41], and BaSCA (Bacterial Single-Cell Analytics) [42–45], a complete pipeline developed by our group for analyzing dense bacterial single-cell movies. The BaSCA pipeline source code is available from GitLab [46].

Currently, the available software tools concentrate mainly on the image analysis tasks, providing only rudimentary analytics capabilities to the user-researcher who may want to model and interpret the sophisticated cell-level dynamics of a complex single-cell movie with multiple interacting colonies and thousands of cells in the microscope’s field of view [47]. Recently, SuperSegger’s GateTool [48] and BactMAP [49] provide the users with advanced post-processing and analytics capabilities. Specifically, both tools mainly focus on visualizing intracellular fluorescence using information from the image itself and subcellular localization of spots and objects, and subsequently on creating plots which can be used in the analysis of time‐lapse movies. However, no tool yet provides vertical analysis and advanced visualization that cuts across all levels of a cell community’s organization. To model the biological processes involved, as cells grow, divide and interact in a dense bacterial community, we should correlate information at multiple scales, from whole cell populations to their interacting colonies and down to the single-cells, and visually and flexibly explore the analytics results.

In this work we present methods for Visualization and Single-Cell Analytics that we developed and implemented as part of a flexible R package [50] (ViSCAR). ViSCAR is provided as an open-source software tool to the research community (see https://gitlab.com/ManolakosLab/viscar). A remarkable feature of ViSCAR is its flexibility. It can be used to analyze single-cell data sets generated either by the BaSCA bioimage analysis pipeline [42–45] or by other popular software packages, such as the Oufti [37], SuperSegger [40], or even Individual Based Modeling (IBM) tools such as the Cellmodeller [51] used to simulate synthetic microbial communities.

All analytics methods presented were enabled by building first an abstract representation of an evolving cell community as a Forest of Lineage Trees (FLT), allowing the storage of cell attributes and the meta-analysis of complex single-cell movies with many interacting colonies. In essence, we transform the useful information of a complex single-cell movie (video) into a hierarchical FLT digital representation and a set of methods, which, when combined, can act as a “digital twin” [30, 31] replica of the live imaging experiment captured by the movie. This rich representation allows us to visualize cell attribute trends easily and intuitively. It also facilitates modeling at a certain level, or across different levels, of community organization (whole cell population, cell colony, generation, cell relatives in consecutive generations) and inferring best mathematical representations (choosing from a variety of parametric distributions) to characterize stochastic phenomena without losing sight of the interactions that may drive them [5–7, 15]. Users may also use ViSCAR to spot and correct inevitable segmentation and tracking errors introduced by image analysis pipelines. All these capabilities of ViSCAR empower research towards deciphering microbial community dynamic behaviors under different environmental and stress conditions.

The rest of the paper is organized as follows. In “Implementation” section, we provide an overview of the implementation of ViSCAR as an R package. In “Results” section, we introduce the basic ViSCAR functionalities using a simple single-cell movie image-analyzed with SuperSegger [40]. Then, we perform a more extensive analysis based on an overcrowded single-cell movie [13] image-analyzed using BaSCA [42]. Finally, we use a very complex synthetic single-cell movie generated using the Cellmodeller tool [51] to demonstrate the importance of predictive analytics in characterizing the stochasticity and investigate how it may affect a large bacterial community’s dynamic behavior. In “Conclusions”, we summarize our findings and point to future directions. In Additional file 1, we include some additional information about ViSCAR package functionalities and some additional results for each case study. In the repository https://gitlab.com/ManolakosLab/viscar, we provide the ViSCAR software R package and the datasets, along with installation instructions and complete documentation. Moreover, in the same repository we provide three R notebooks that one can run after installing ViSCAR to reproduce all the results in “Results” section, and a tutorial video on Case Study I that one can watch in order to start testing ViSCAR’s capabilities.

## Implementation

### The ViSCAR architecture

ViSCAR is a set of methods and their R package implementation for the Visualization and Single-Cell Analytics of data sets derived from the bioimage analysis of complex time-lapse bacterial single-cell movies. It enables analytics of single-cell data sets at different levels of granularity (image frame, cell subpopulation, colony, generation, etc.), provides many ways to analyze and visually explore the spatio-temporal evolution of cell attributes, can be used to study trends, compute correlations, and estimate best-fit model parameters, such as single-cell growth and kinetic parameters, to support predictive modeling for systems biology.

**Fig. 1:**
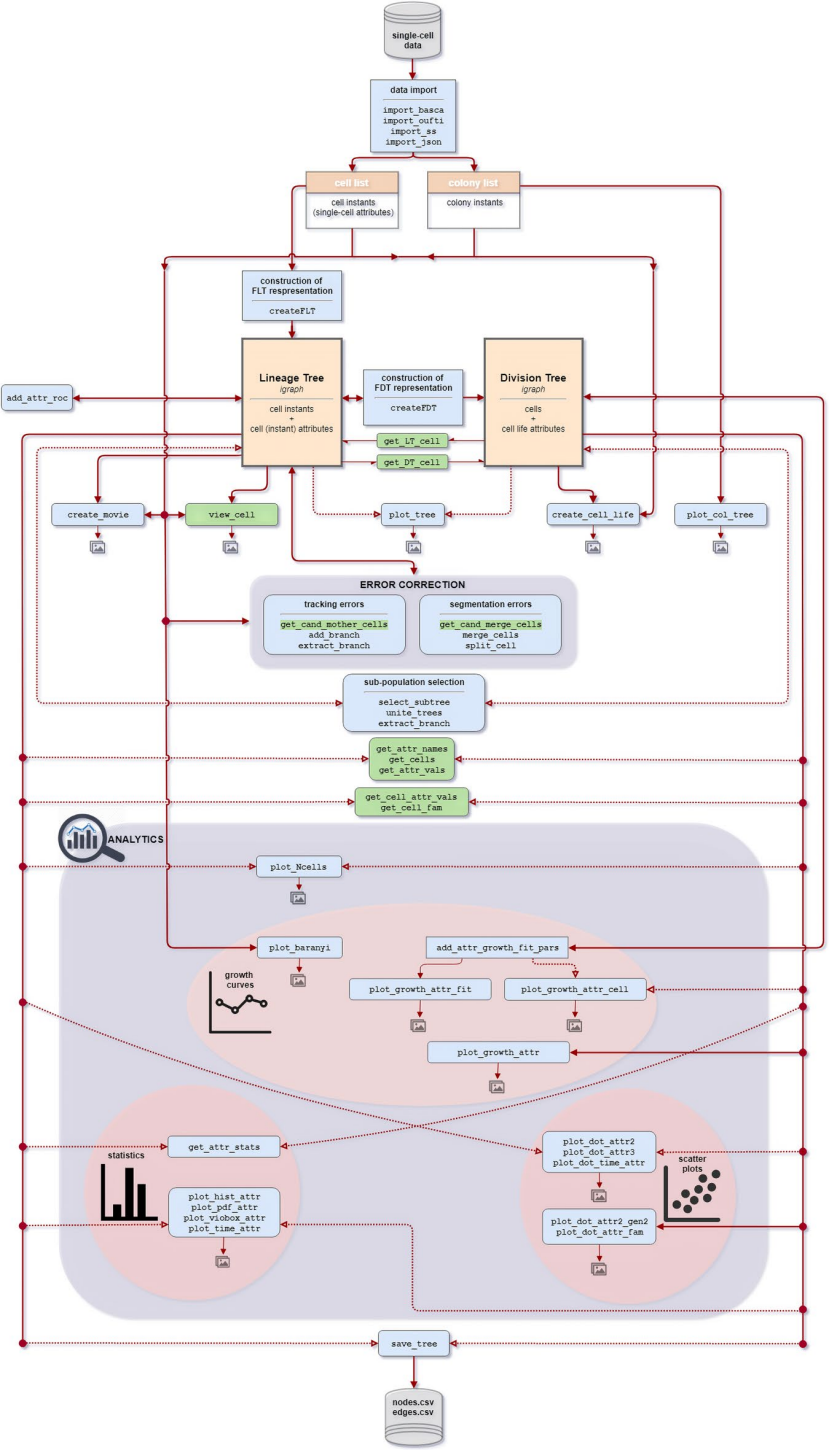
Overview of the ViSCAR architecture. Orange boxes represent data structures; blue boxes correspond to the core functions; green boxes represent useful auxiliary functions. Analytics capabilities (blue area) are organized into three categories: estimation of growth curves, statistics, and scatter plots. Solid lines mark the input/output data structures of functions. Dotted lines are used for alternative input structures. An image icon output is shown for functions generating plots

Figure 1 provides a top-to-bottom overview of ViSCAR’s architecture. The R implementation mainly depends on the *igraph* [52] package for the tree representation of the single-cell movie, and the *ggplot2* [53] package for the majority of visualization functionalities and utilizes functions from other R packages as well [54–63]. A full description of ViSCAR functions is provided in the package’s documentation (see https://gitlab.com/ManolakosLab/viscar).

### Data importing

ViSCAR supports various input file formats for importing data sets of analyzed single-cell movies. It offers functions for automatically importing the single-cell data exported by BaSCA [42–44], Oufti [37], and SuperSegger [40]. These functions convert the input file(s) into a cell list (and colony list), containing all the cell instances (and colony instances) of the single-cell movie. Besides, custom user-constructed cell (and colony) list structure(s) saved in.json file format can also be directly imported, provided that they have a prespecified form described in the package’s documentation (see https://gitlab.com/ManolakosLab/viscar).

### The forest of lineage trees representation of complex single-cell movies

**Fig. 2:**
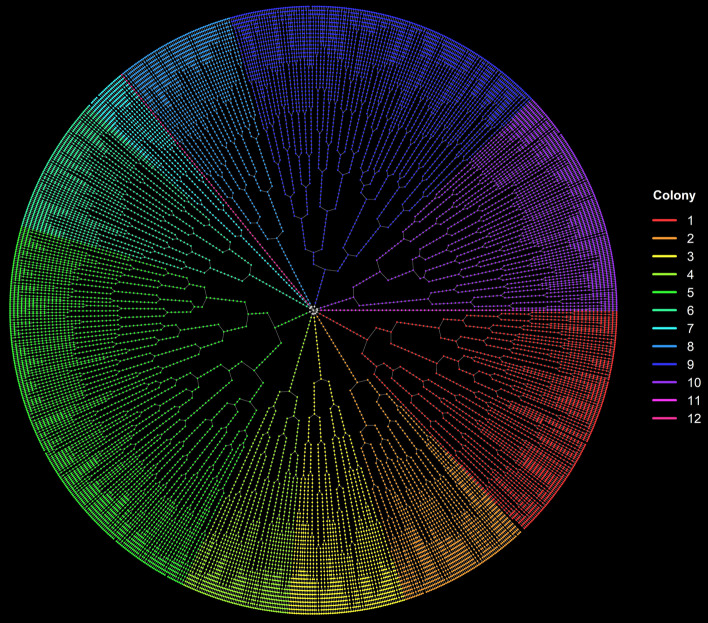
Forest of Lineage Trees (FLT) representation of the complex multi-colony single-cell movie. The colony ID is used to color the cell instances. Nodes that do not correspond to cells (central “root” and colony “roots”) are uncolored. Each tree level represents a frame of the movie (80 levels, 78 frames, 5 min frame rate)

Once the cell list of the single-cell movie is loaded (see Fig. 1), we use the createFLT function to transform it into a Forest of Lineage Trees (FLT) data structure, storing a summary of all cell’s information at all time-points (cell instances). The FLT is a set of lineage trees (LT), with each LT representing a different colony in the single-cell movie. A lineage tree node represents a cell instance, i.e., an individual cell at a specific frame (a time point of its lifespan). Only the FLT master root node (at the center of the circle) and each colony’s root nodes do not correspond to actual cell instances. Therefore, the first movie frame’s cell instances are the daughters of the corresponding colony’s root node and appear at level 3 of the FLT. So, levels n ≥ 3 of the FLT represents the actual frames of the single-cell movie. The FLT and colony root nodes are included to facilitate the movie’s abstract tree representation and colony tracking and are excluded from the data analysis. A typical FLT of a complex single-cell movie is shown in Fig. 2.

The life of a cell is delineated by two events: cell birth and cell division. We define as birth and division times, the frames (time points) at which the cell is firstly and lastly spotted in the single-cell movie. In bacterial image analysis, a cell division event, i.e., cell disappearance from the field of view, may result from an actual cell division, a cell death, an exit from the microscope’s field of view, single-cell movie termination, or a cell tracking error. Similarly, apart from a cell division event, a cell birth event may arise when a cell enters the field of view or when a cell failed to be associated with any cell instance in the previous frame (cell tracking error). To ensure that only real newborn cells emanating from actual cell divisions contribute to the data analysis, we store the “motherless” FLT branches separately. As we will demonstrate in “Image analysis error spotting and correction” section, motherless branches arising from tracking errors can be attached by the user to the correct FLT node using the error correction capabilities of ViSCAR.

**Fig. 3:**
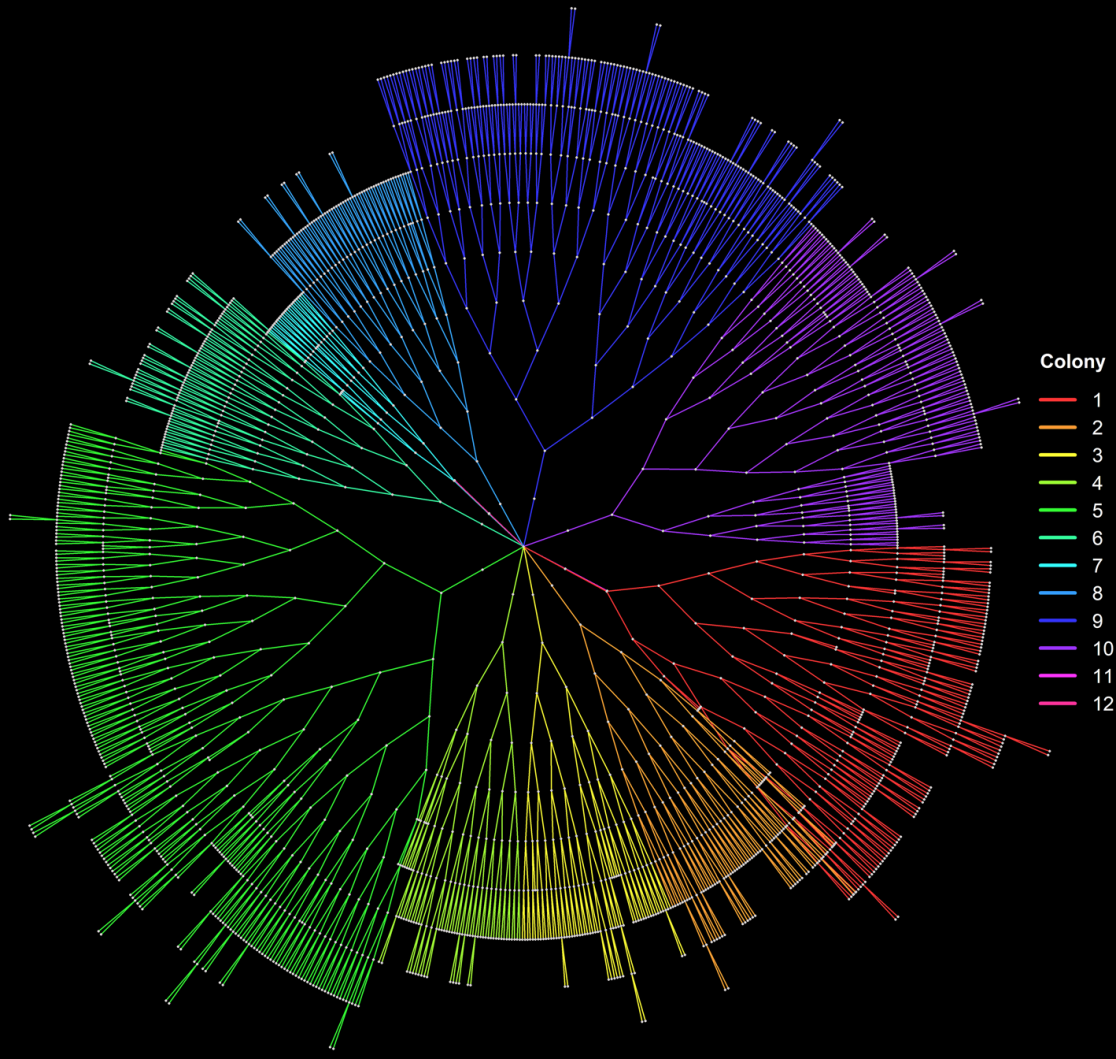
Forest of Division Trees (FDT) representation of the complex multi-colony single-cell movie. Subtree colors denote the corresponding colony. Each level of the tree represents a cell generation (13 levels, 11 generations). Notice that colony 11 and 12 do not grow; they remain single-cells. Thus, they are represented by single-node branches near the FDT’s main root

A segment (sequence) of LT nodes between a birth and a division event represents a cell’s lifespan. Suppose we reduce a LT cell segment (lifespan) down to a single node. In this way, we obtain the Forest of Division Trees (FDT) single-cell movie representation and data structure, capturing in a node only each cell’s division event and summarizing its lifespan. Just like the FLT, the FDT also contains the master root and the colony root nodes. Level n ≥ 3 of the FLT represents the (n–3) generation of cells in the movie since the generations index starts counting from 0. The FDT of a complex single-cell movie is shown in Fig. 3. The data analysis can be automatically restricted to cells observed in the single-cell movie for their complete lifespan (i.e., from their birth and all the way to their division event) or for an incomplete lifespan (i.e., from their birth and for at least a minimal number of frames before their division). We can achieve this by excluding cell trajectories terminating in leaf nodes or with a length less than a minimum user-defined number of frames.

The FLT and FDT are the core data structures used to represent complex single-cell movies and are modeled as objects of class *igraph*. *Igraph* [52] is an R library designed for the fast handling of large graphs with millions of vertices and edges. It provides a set of data types and functions suitable for the FLT/FDT representation of a single-cell movie, allowing the storage of node (vertices) attributes, selecting nodes based on their attribute values, and visualization of a tree. The importance of these facilitations will be clarified in the following sections.

### Cell attributes

We have two categories of the single-cell attributes: cell “instance attributes” (or just “attributes”), which may change their value at each time point (frame), and cell “life attributes,” that characterize a cell’s whole lifespan.

**The cell (instance) attributes** are estimated by image-analysis software. For example, they may include the cell area, the major/minor cell axis length, the cell distance from the colony’s centroid, the mean fluorescence value, whether the cell is located at the boundary of its colony touches other cells, etc. These phenotypic or expression, numeric or Boolean, attributes are loaded into the cell list and finally stored as node attributes in the FLT representation of the single-cell movie with the createFLT function call. Other features computed by ViSCAR, such as the cell age or the instantaneous rate of cell length change, are also cell attributes.

**Cell life attributes** are estimated using the createFDT function and are stored as node attributes in the Forest of Divisions Tree (FDT). These attributes are the birth time, division time, and basic statistics (min, max, mean, standard deviation) of every numerical cell attribute, characterizing the entire cell life. For instance, such life attributes are the average cell length (from cell birth to cell division), the division time, length at division, area at birth, etc. Each boolean cell attribute can also be reduced to a corresponding cell life attribute (with value TRUE or FALSE) based on the majority reduction of the collapsed LT nodes’ corresponding attribute values. In addition, given each cell’s time-series data for a numerical attribute (e.g., the cell length), the package can estimate single-cell growth parameters (e.g., the cell elongation rate) by fitting a linear or exponential model to this lifespan time series data of each individual cell (see “Case study I: Basic single-cell analytics workflow” section for details).

In order to support analyses at the colony or generation levels automatically, the colony and generation information of each cell is stored as attribute in both the LT and DT nodes for the cell. Note that the “colony” attribute points to the colony from which each cell (instance) has emanated. This approach is necessary to keep track of a cell’s origins even after two colonies have merged.

### Subpopulations selection

Users can isolate cell subpopulations of interest for closer analysis. Using the select_subtree function, users can select a lineage or division tree subpopulation based on multiple constraints. A constraint may involve an attribute, a comparison operator, and a threshold value. The attribute can be any numerical or boolean cell attribute stored in the LT or cell life attribute stored in the DT. Selection constraints can be combined with logical AND operators, equivalent to sequential selections on the initial tree population. To connect multiple selection criteria with logical OR operators (i.e., for uniting different subpopulations), the unite_trees function can be used.

These capabilities enhance flexibility in subpopulation selection and promote the ability to perform ad-hoc analysis. For instance, it is straightforward to limit the analysis to a specific subpopulation of interest, e.g., cells with average fluorescence intensity within a particular range, in specific colonies or generations. Note that the selected subpopulation of a lineage or division tree is also considered a tree structure and can be used as input to other functions, even though it may ultimately become an unconnected graph. Selection operations are also useful in removing cell debris and identifying segmentation and tracking errors, as described in 2.8.

### Single-cell analytics at multiple levels of community organization

Our single-cell analytics approach enables zooming-in and extracting useful information regarding the subpopulation characterization for any cell (life) attribute of interest. Users can create scatter plots, estimate growth curves, and perform statistical analysis of single-cell attributes at any community organization level (all cells, subpopulation, selected colonies, generations, subtrees of individual colonies, etc.), in space or in time. The full list of the related functions is provided in Additional file 1: Table S1. These flexible and insightful analytics capabilities allow users to formulate interesting new hypotheses for future research.

#### Scatter plots

Scatter (or dot) plots are essential tools of exploratory data analysis. We offer various functions for generating scatter plots [64] of numerical single-cell attributes, allowing the user to study their correlations.

As indicated in Fig. 1 (bottom right), the name of these functions starts with the prefix “plot_dot_ <  > ” followed by a string denoting the displayed variables. For example, plot_dot_attr2 and plot_dot_attr3 create the XY or XYZ scatter plot of two or three attributes, respectively. plot_dot_attr2_gen2 creates the XY scatter plot of the same or different attributes between cells of two different cell generations. plot_dot_attr_fam creates the XY scatter plot of an attribute between cells derived from the same ancestor (i.e., siblings, cousins, mother and daughter, or grand-mother and grand-daughter cells). Lastly, the plot_dot_time_attr function creates several dot plots of a cell attribute, one dot plot per time point (movie frame) or per generation index, that are similar to box plots but with no whiskers. They can be used to visually assess how the variance of the examined cell attribute (e.g., cell length) evolves with time.

The data is displayed as a collection of points on XY axes. If the points are color- and/or shape-coded, one or two additional attributes can be displayed. In some functions, color is also used to denote the density of the data by binning and counting the number of points in each bin. In this way, we can avoid overplotting (i.e., distinguish how many points are plotted at each location) and, at the same time, visualize a color representation of the histogram of the displayed attribute(s).

Depending on the generated scatter plot dimensions (XY or XYZ), the linear regression line or plane [64] is also drawn. The equation of the regression line/plane is determined using the linear least-squares method. The estimated parameters and the R-squared coefficient [64, 65] of the regression line/plane are returned to the user. For XY scatter plots, the Pearson correlation coefficient is also computed.

#### Growth curves

The user can also compute growth curves for the whole population, or each colony cell counts, by fitting the primary model of Baranyi and Roberts [66] to the data using the non-linear least-squares method [67]. Microbiologists commonly employ this mathematical model to estimate a cell population's kinetic growth parameters in a given environment.

Given the values of a cell attribute at every time instance and using the add_attr_growth_fit_pars function, we can also fit each cell trajectory (time series of an attribute) to a linear or exponential growth model [68, 69] using the linear or non-linear least-squares method [67], respectively. In this way, we can capture each cell’s growth characteristics, estimate its *personalized* kinetic parameters, and save them as cell life attributes in the DLT. The root means squared error (RMSE) of the fit is also computed and can identify abnormal growth cells.

The package offers several functions for the visualization of the single-cell growth curves. For instance, we can plot the unfitted single-cell growth curves of an attribute from the start to the end of the movie, or the fitted single-cell growth curves of an attribute for a specified period, per colony or generation, colored by cell life attribute (e.g., cell division time). These capabilities allow us to observe and characterize the single-cell growth kinetics’ variability, which population-based experiments cannot observe.

#### Stochasticity analysis

In addition to computing basic statistics (mean, median, standard deviation, min, max), ViSCAR offers the capability to quantify the stochasticity and examine inter- and intra-colony and inter- and intra-generation variability of single-cell attributes. We can visualize the time evolution of an attribute’s mean and standard deviation, create violin plots, box plots, histograms, and even estimate the average fitted single-cell growth curves.

Moreover, we can compute and plot the Probability Density Function (PDF) of any cell (life) attribute of our choice by fitting Normal, Lognormal, or Gamma distribution models to the data using maximum likelihood estimation (MLE) [65]. The best fit parametric model among the ones mentioned above can also be automatically estimated based on the Bayesian Information Criterion (BIC). We do not limit the analysis to the normal distribution since many biological mechanisms may induce skewed (Gamma, Lognormal) distributions (e.g., the cell division time [70, 71], cell division length [69], and the cell elongation rate [71]).

All these functionalities can be performed for the whole cell population in a tree, per colony, or per generation. Color in these plots denotes the corresponding colony or generation and is consistent throughout the single-cell movie dataset analysis. The axis range of the depicted attribute is common, allowing comparisons between the specified colonies or generations and assessments of the exhibited heterogeneity.

### Visual analytics capabilities

#### Visualizations

Our methods allow the user to visualize the evolution of single-cell attribute values in two ways. Using the plot_tree function, we can exploit the FLT (FDT) construction and depict an extracted/computed cell instance (life) attribute using color on top of tree nodes. For example, we can map the single-cell area on a lineage tree’s nodes and visually examine how it changes during the cells’ lifespan. We can also map the cells’ division time on a division tree and assess how it evolves across generations. A unique feature that, to the best of our knowledge, no other framework provides is to use the create_movie function and generate time-lapse movies of the segmented cells, animating the visualization of any selected cell (life) attribute. These visual analytics features are beneficial since they can help users quickly capture an attribute’s variability across cells, colonies, frames, or generations. We provide two animated single-cell movies, in Additional files 2 and 3, visualizing the single-cell area (a cell instance attribute) and single-cell colony ID (a cell life attribute) of a single-cell movie.

#### Figure exporting

Functions starting with the prefix “plot_ <  > ” create plots for either visualization or analytics purposes (see functions with an image icon in their output in Fig. 1). The plots are by default displayed in the Plots Pane of RStudio [72] and can be exported as images or PDF files through the Export option. The drawback of the RStudio export is that the images are screenshots of the plots, having a defined resolution. ViSCAR allows users to save the generated figures of each function in.png file format for external use and specify the width, height, and resolution of these images.

### Image analysis error spotting and correction

Near perfect cell segmentation and tracking are prerequisites for obtaining correct FLT and FDT representations of single-cell movies and, consequently, accurate meta-analysis results. However, the image analysis of complex single-cell movies is a difficult task. Most state-of-the-art software’s output requires a significant amount of human effort for manual curation due to errors caused by imperfect segmentation and tracking algorithms. Even the identification and location of these errors remain tricky for the inexperienced eye.

Our method allows users to detect and correct cell tracking errors by manipulating the FLT, adding to and(or) removing branches, while also visualizing the questionable cell neighborhoods. These capabilities are offered by the add_branch and extract_branch functions, respectively. In addition, segmentation errors can be corrected by merging or splitting under- or over-segmented cells, respectively. These operations of merge_cells and split_cell functions are performed by calling a MATLAB [73] executable, which should be separately installed by the user. This executable is based on BaSCA algorithms and requires the.mat file exported by the BaSCA image analysis pipeline [42–44].

**Fig. 4:**
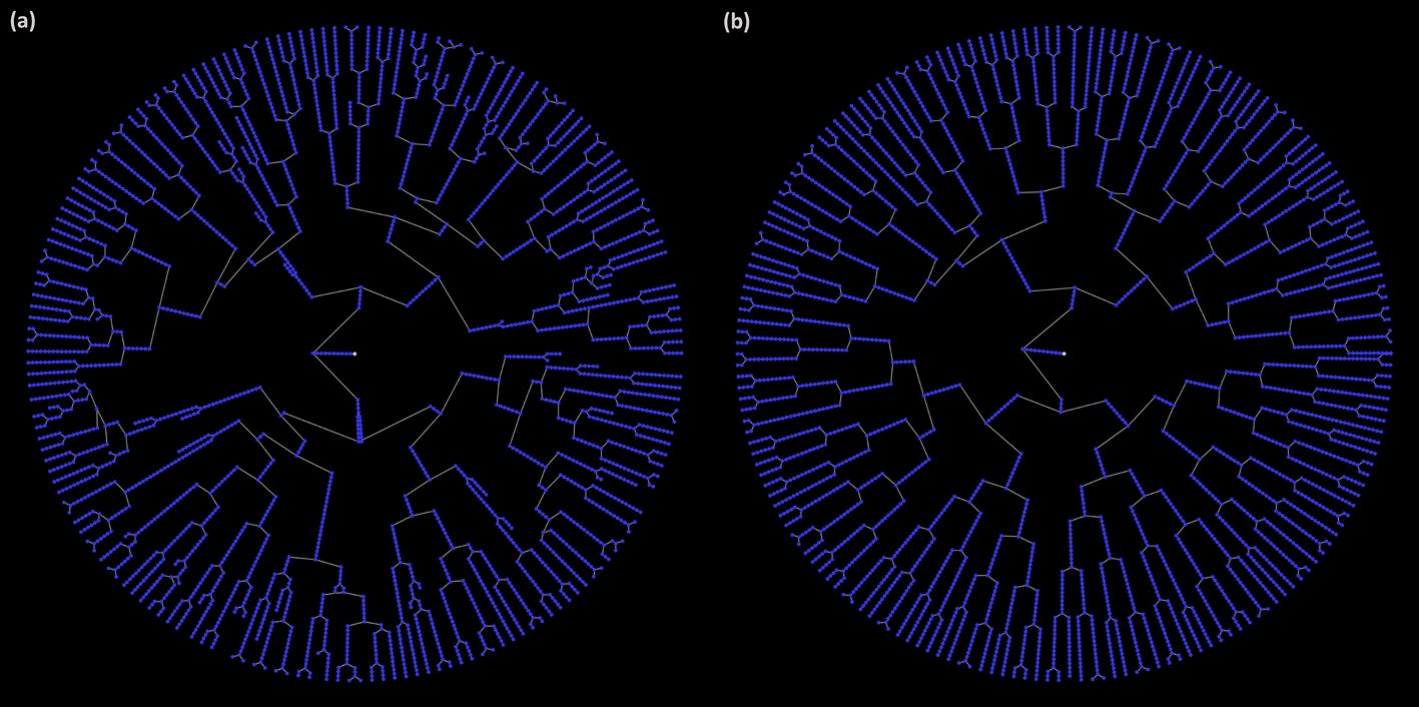
A lineage tree **a** before and **b** after error correction. Each node represents a cell instance of a colony growing in a single-cell movie. Notice that in (**a**) there are branches that do not reach the LT’s last level (i.e., there exist leaf nodes at several levels). In single-cell movies with no cell death events, this tree pattern cannot occur. In the corrected LT of (**b**), all subtrees reach the last level as expected

**Fig. 5:**
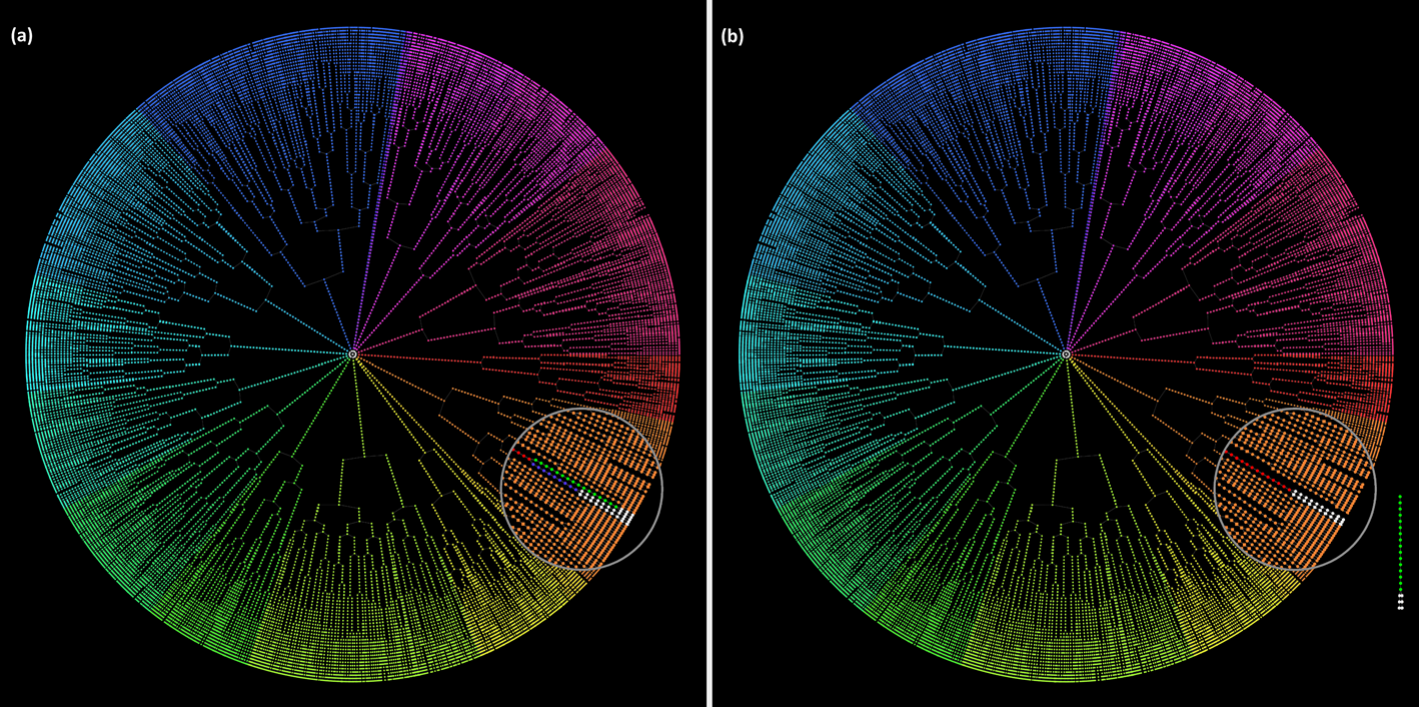
FLTs of a single-cell movie: **a** Correct, and **b** erroneous versions. Colors represent different evolving colonies of the time-lapse single-cell movie. **a** In the magnified area, we observe a red cell that divides, giving birth to two daughter cells, a green and a blue. **b** In this incorrect version, the red cell’s division event was missed, and an unmatched isolated branch appears to the far right. The correct FLT of (**a**) was recovered from the one in (**b**) by using the add_branch function to “glue” the isolated branch under the 4^th^ red node of the red cell trajectory, thus recovering the missed division event

Image analysis errors often lead to an unbalanced FLT (see Fig. 4). They may occur due to the failure of the tracking algorithm to match two cells in consecutive frames. This results in motherless branches that our algorithms automatically spot and store separately (see Fig. 5). Therefore, a first step towards correcting and extending the FLT is “gluing” such motherless branches to their correct position.

**Fig. 6:**
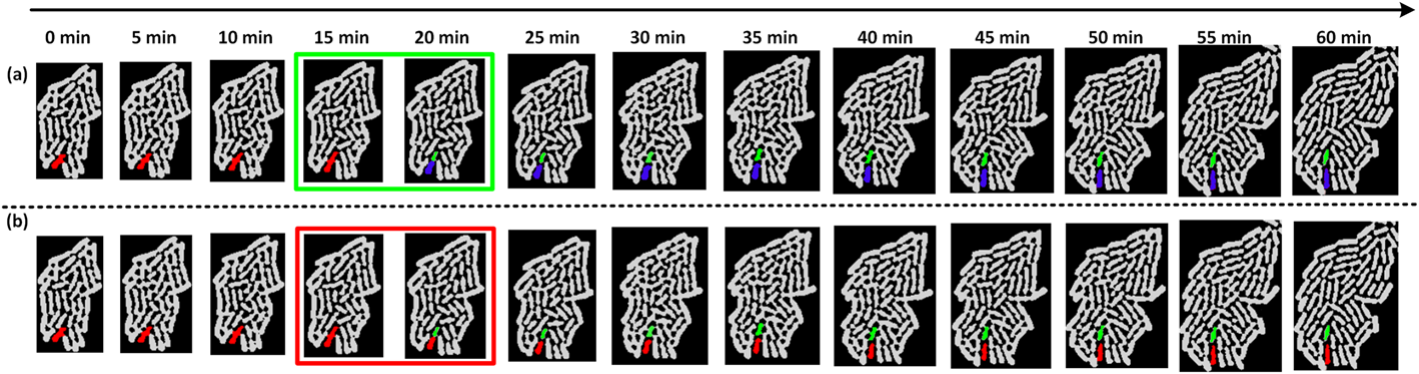
Cell life visualization. The user can monitor how a selected cell life evolves in the single-cell movie and detect tracking or segmentation errors. The chosen cell here is the red cell in the magnified area of Fig. 5. In **a**, corresponding to the correct FLT of Fig. 5a, the red mother cell divides between t = 15 min and t = 20 min as noted in a green box, i.e., the red trajectory terminates at t = 15 min and two daughter cells (green and blue) emerge in its space at t = 20 min. The red cell instances up to t = 15 min correspond to the 4 red nodes in the red subtree of Fig. 5a. After the division event, the green and blue cell instances correspond to the 9 green and 9 blue nodes of the respective subtrees in Fig. 5a. In **b**, corresponding to the FLT of Fig. 5b, we observe the missing red cell division (red box); the red cell trajectory continues, along with only one (green) daughter cell, after t = 15 min while the blue cell is missing. The red cell instances correspond to the 13 red nodes of the incorrect subtree in the magnified area of Fig. 5b. The green cell instances correspond to the 9 green nodes of the unmatched isolated branch shown in the right bottom part of Fig. 5b

In Fig. 5, we present a correct FLTs (left) and an incorrect (right). Although the FLT may be balanced and seemingly correct, errors may still remain uncovered (see magnified part of Fig. 5b). These errors can be identified by monitoring how each cell life evolves in the single-cell movie (see Fig. 6). The create_cell_life function offers this capability for a selected cell. In Figs. 5 and 6, we present how the create_cell_life and add_branch functions can be used in combination to correct a hidden tracking error.

In the magnified area of Fig. 5a, we can see a red cell that evolves and eventually divides, giving birth to two daughter cells, a blue and a green cell. In the magnified area of the incorrect version, Fig. 5b, we can see that the red cell did not divide. The missing division event implies that the tracking algorithm failed to match the mother cell instance with the two daughter cell instances. An unmatched branch appears that must be glued to the appropriate red cell instance to recover the missed division event. By inspecting selected cell lives, using the create_cell_life function, we can observe the green cell life in Fig. 6b, and understand that there is a missing division event because the green cell has no mother. By checking its neighing cells, we can spot the red cell that should be its mother because it has an anomaly in its trajectory; it grows, then its area drop abruptly, and then increases again. We can correct the FLT by using the add_branch function upon identifying the unmatched green cell and its mother, the red cell.

Methods to efficiently focus on specific cell lives and their neighborhoods are necessary to support active live-cell microscopy. Spotting cell instances that present certain anomalies allow specialists to locate cell phenotypes of interest in an overcrowded colony near real-time.

Yet, the time required to inspect and separate all accurately segmented/tracked cells from inaccurately segmented/tracked ones is prohibitive, especially for complex single-cell movies with thousands of cells. Since cells typically exhibit exponential length growth (elongation) [68, 69], this can help us formulate an effective strategy for identifying cells with segmentation and tracking errors. Such cells tend to have length curves that either fail to fit an exponential growth model or significantly deviate from it (i.e., have an RMSE above a specified threshold). In the latter case, we can choose an appropriate threshold by creating the histogram of all cells’ RMSE values in the movie. Therefore, we can easily spot potential errors on the FLT by selecting these cells and visualizing their lifespans.

Another approach for quickly spotting problematic cells is to monitor how the cell length or cell area changes with time. Typically, cells with a significant increase or decrease of these attributes from frame to frame are incorrectly segmented or tracked. Therefore, computing the instantaneous rate of change (roc) for the length (or area) makes detecting such cells easy. These descriptors can be estimated for every cell instance in the FLT by calling the add_attr_roc function. They can also be normalized to provide the change percentage.

## Results

### Case study I: Basic single-cell analytics workflow

In this case study, we introduce the basic workflow of a single-cell movie dataset analysis using ViSCAR. We will use a dataset as input to highlight ViSCAR’s capability to process the results produced by an image analysis software other than BaSCA.

#### Dataset 1

This simple time-lapse phase-contrast optical microscopy single-cell movie shows the evolution of a single microcolony of Escherichia Coli with 12 progenitor cells. The genus Escherichia comprises rod-shaped bacteria with a length of ~ 2 μm and width ranging from 0.25 to 1 μm [74]. The single-cell movie consists of 60 frames, acquired with 1 min sampling period and 362 × 331 pixels resolution. The movie and its analysis are available on SuperSegger’s webpage [75]. The dataset needed to reproduce all results of this section is included in https://gitlab.com/ManolakosLab/viscar.

#### Dataset exploration

**Fig. 7:**
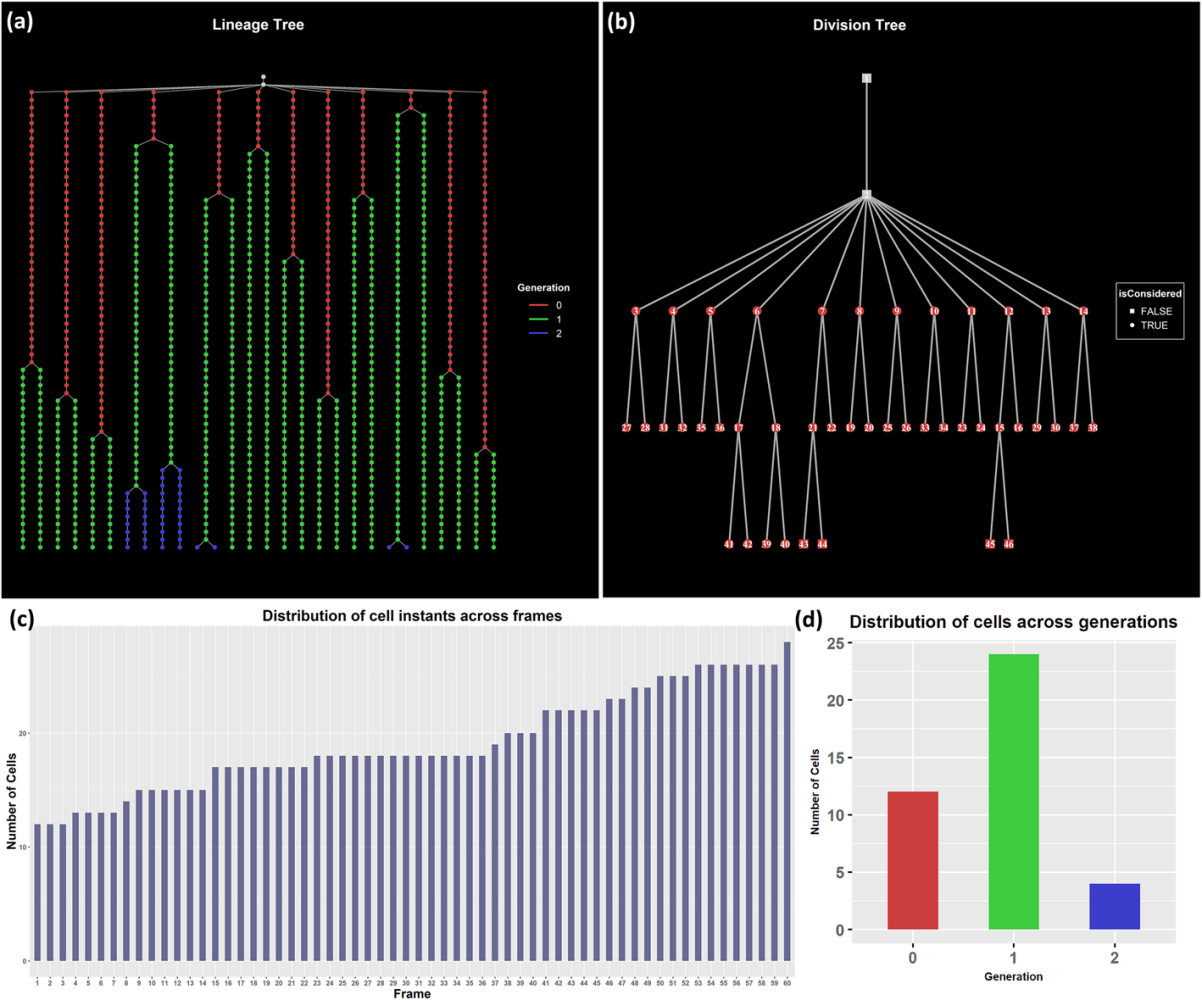
Typical plots for a general overview of dataset 1 single-cell movie. **a** LT representation of the movie. Each level of the tree represents a frame of the movie. Cell instances are colored by generation. Main “root” and colony “root” nodes are uncolored. **b** DT representation of the movie. Each level *n* of the tree with *n* ≥ 3 represents a cell generation. The cells of the movie are colored red. Main root and colony root nodes are uncolored. **c** The number of cell instances per movie frame (LT level). **d** The number of cells per generation (DT level)

We load the dataset and create the FLT and FDT representation of the corresponding single-cell movie, depicted in Fig. 7a, b, respectively. Note that since SuperSegger treats the whole dataset as a single colony, the FLT consists of a one-colony LT. In Fig. 7a, we observe asynchronous divisions of the cells (i.e., change of their color). However, since we do not know the initial setup of the experiment (e.g., the size of each progenitor cell at the beginning of the movie), this observation is non-informative regarding the cells’ division. In Fig. 7c, d we provide the distribution of the cells across frames (LT levels) and generations (DT levels). Cells that live for less than 5 frames (5 min) are automatically excluded from the analysis.

#### Subpopulation selection

**Fig. 8:**
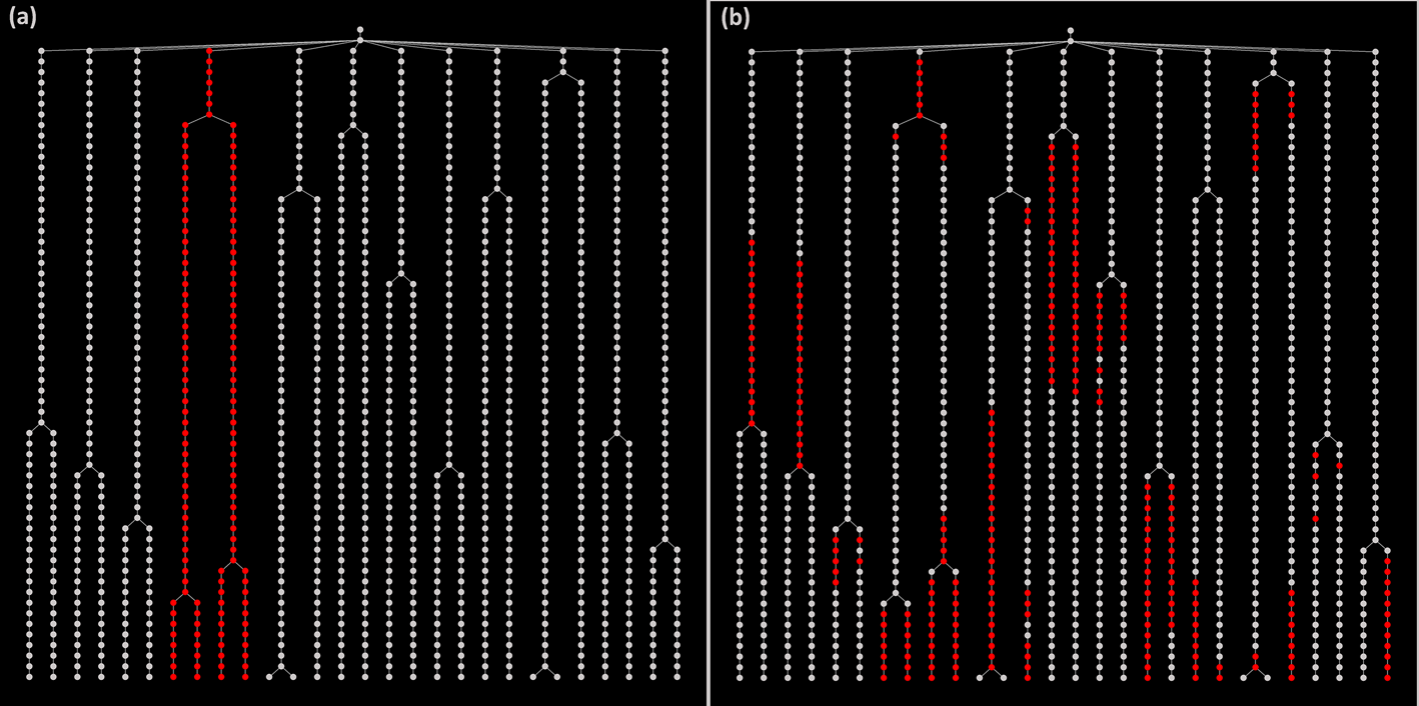
Examples of subpopulation selection and their visualization on the lineage trees. **a** A subtree (red) of a progenitor cell (cell “6” of the dataset). **b** Cell instances (red) contact other cells and are also outliers in terms of their cell area

We can perform elaborate selections of cell(s) (or cell instances) and apply any desirable analysis and visualization on the resulting subpopulation. For example, we can select and visualize on the FLT the lineage tree of a progenitor cell (Fig. 8a), or the cell instances which are in contact with other cells and are outliers in terms of their cell area (Fig. 8b). As outliers, we consider here cells with an area smaller or larger than one standard deviation below/above the mean, respectively (Mean and Standard Deviation Method).

#### Single-cell analytics

**Fig. 9:**
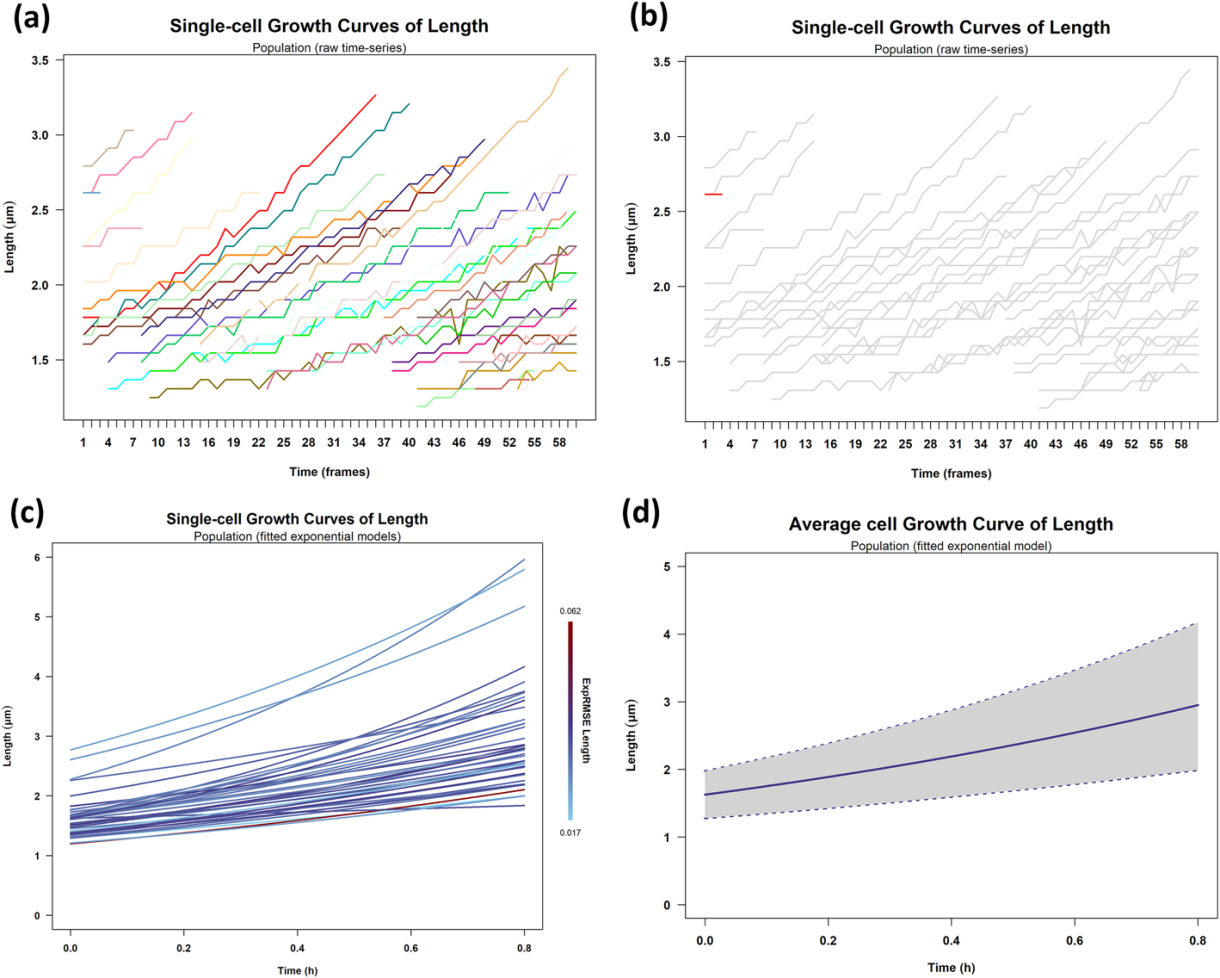
Single-cell growth analytics. Visualization of raw single-cell length growth curves. Each jagged line represents the length time-series of a cell in the population (raw data). Time (x-axis) is the frame index in the movie. **a** Cells were randomly colored. **b** The small length red curve (upper left region) marks the only cell that failed to fit an exponential length growth model. Gray curves depict the rest of the cells. **c** Single-cell length exponential growth curves after model fitting. Each curve represents a cell of the population. Color denotes the RMSE of the exponential fit to the cell’s length. **d** Average and standard deviation of the population’s exponential length growth curve (see text for details)

Users can perform multiple analytics operations at the population and single-cell levels. Given the length of every cell at every time point (frame) (Fig. 9a), we can fit an exponential model to each cell’s length trajectory (time-series) [68, 69] and estimate the cell’s personalized elongation rate. When in the “exp” mode, the plot_growth_attr_fit function fits the exponential model $y = y_{0} \cdot e^{kt}$ to the corresponding cell attribute *y* using the non-linear least-squares method [67] and stores in the FDT the estimated model parameters. When the length attribute is used, *y* represents the cell length, *t* the time, *y*_0_ the cell length at birth, and *k* is the cell (length) growth (elongation) rate. The IDs of the cells that failed to fit the specified model (due to lack of enough time-points) are returned to the user, and their raw data can be inspected (Fig. 9b). Figure 9c shows the exponential growth curves of each cell using the plot_growth_attr_fit function. The function also plots the average exponential growth curve of the population $\overline{y} = \overline{y}_{0} \cdot e^{{\overline{k} \cdot t}}$ (solid line in Fig. 9d), and marks the variance around it (gray area in Fig. 9d). Two lines define the variance; $\overline{y}_{ \pm } = \left( {\overline{y}_{0} \pm s_{{y_{0} }}^{2} } \right) \cdot e^{{\left( {\overline{k } \pm s_{k}^{2} } \right) \cdot t}}$ (upper and lower dotted line). The estimated parameters of the lines are returned to the user. For this single-cell movie, the estimated parameter values are $\overline{k } = 0.748\,\upmu {\text{m}}/{\text{h}}$, $s_{k}^{2} = 0.190$, $\overline{y}_{0} = 1.644\,\upmu {\text{m}}$ and $s_{{y_{0} }}^{2} = 0.350$. We observe that the same colony cells may exhibit great variability in their growth kinetics, a known phenomenon masked in population-based experiments. Please refer to Additional file 1: Section 2 for more examples on single-cell analytics of Case Study I.

### Case study II: Complex single-cell movie analytics and visualization

In this case study, we present a more elaborate workflow using dataset 2. The dataset was produced by applying BaSCA-based image analysis to a complex single-cell movie. We use it here to highlight the advanced capabilities of VISCAR, and how they can be instrumental in characterizing how single-cell attributes vary across colonies and evolve along cell generations in a growing cell community.

#### Dataset 2

This time-lapse phase-contrast optical microscopy single-cell movie depicts multiple growing microcolonies of *Salmonella enterica serotype Typhimurium*. The genus Salmonella, which is closely related to the genus Escherichia, comprises rod-shaped bacteria ranging in diameter from 0.7 to 1.5 μm, with a length of 2 to 5 μm [74]. The single-cell movie originally consists of 101 frames, acquired with 5 min sampling period and 1360 × 1024 pixels resolution (see [13] for details). It shows the simultaneous growth of 21 bacterial clones, each emanating from a single progenitor cell, which eventually are merging (starting from the 27^th^ frame). We performed the image analysis using BaSCA since all other state-of-the-art software we tried failed to produce satisfactory results for this complex single-cell movie. To obtain reliable analytics results (see “Case study II: Complex single-cell movie analytics and visualization” section), we restricted our analysis to the movie’s first 78 frames and only 12 colonies. We have high confidence that these were correctly segmented and cell-tracked. This part of the single-cell movie contains overcrowded merging colonies with more than 19,000 cell instances in total, exceeding 1000 in the last frame. Overall, there are 1695 growing cells, with an average length of 2.374 μm and an average width of 0.798 μm. The dataset needed to reproduce all results of this section is included in https://gitlab.com/ManolakosLab/viscar.

#### Dataset exploration

**Fig. 10:**
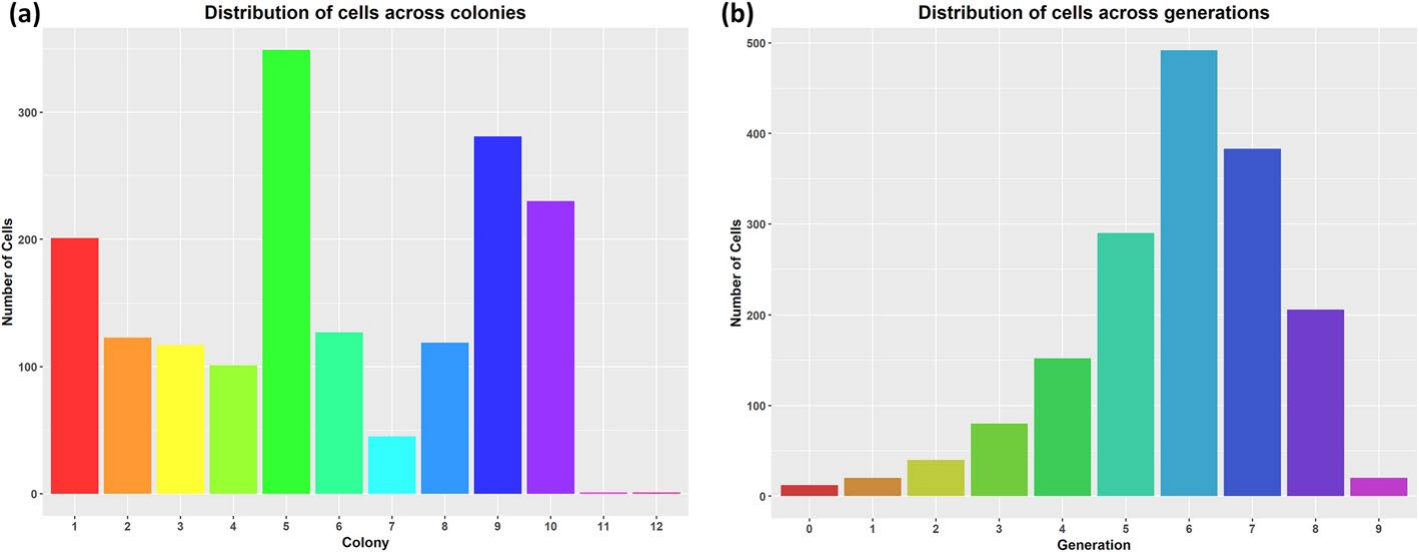
Histogram of cells included in the analysis across **a** colonies and **b** generations

We first load the dataset and create its FLT and FDT representations. In Fig. 10, we provide the distribution of the cells across colonies and generations. Cells that live for less than five frames (25 min) are automatically excluded from the analysis. The FLT and FDT representations of the movie are shown in Figs. 2 and 3, respectively. The large differences observed in the number of cells per colony and per generation (Fig. 10) lead to an unbalanced FDT (Fig. 3). These differences occur due to inter- and intra-colony and generation variability of the cell division time, and they are explored further below.

#### Estimation of colony growth curves

For each colony in the movie, we used the plot_baranyi function to fit the Baranyi and Roberts model [66] to the cell counts growth curve. The equation of the model is:

1 $$y = \log N_{max} + \log \frac{{ - 1 + e^{{\mu_{max} \cdot \lambda }} + e^{{\mu_{max} \cdot t}} }}{{ - 1 + e^{{\mu_{max} t}} + e^{{\mu_{max} \cdot \lambda }} \cdot 10^{{(\log N_{max} - \log N_{0} )}} }}$$

**Fig. 11:**
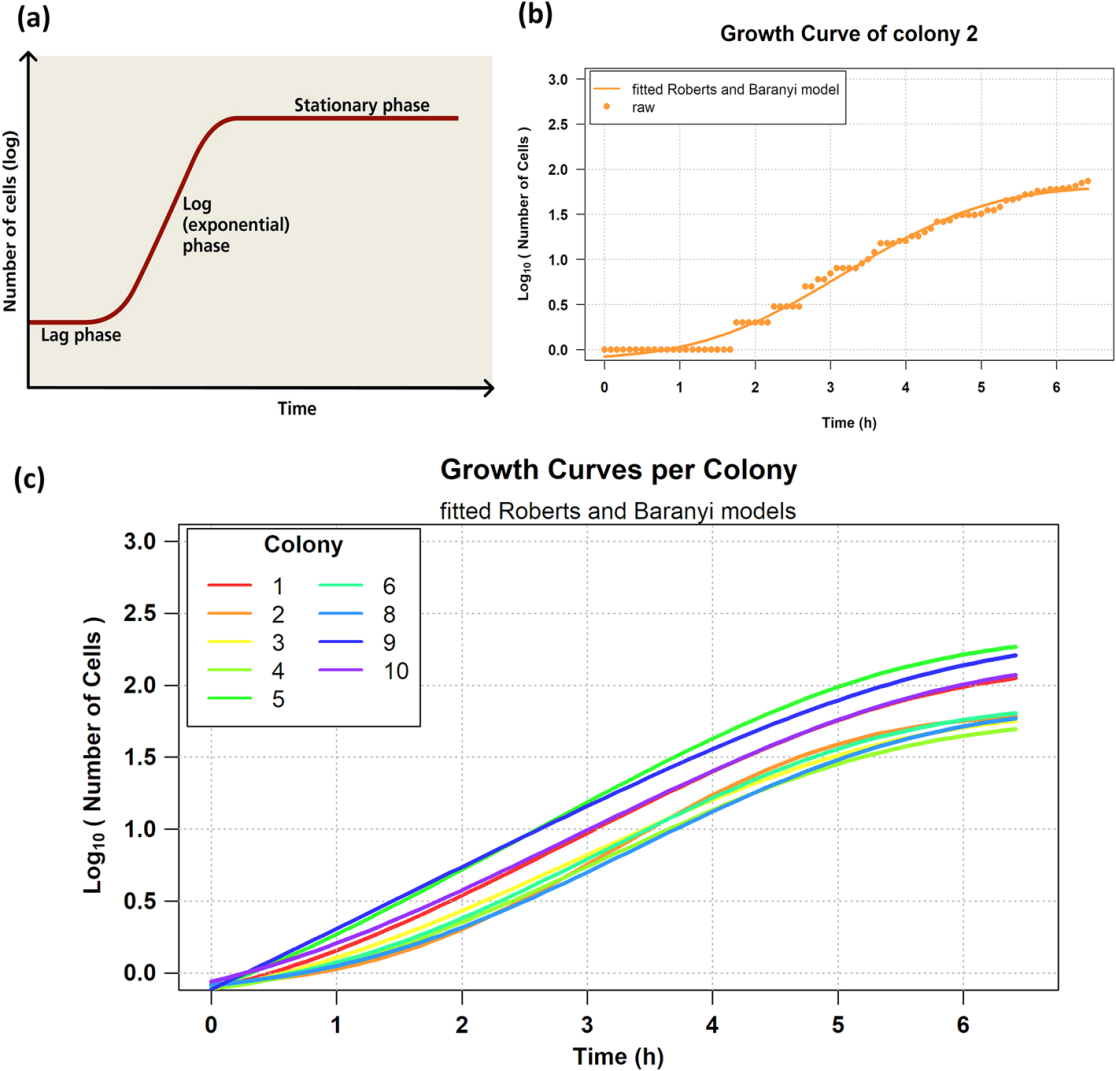
Colony growth curves. **a** The Baranyi and Roberts model [66] represents the three bacterial growth stages: lag, exponential, and stationary. **b** Raw (dots) and fitted (curve) cell counts of colony 2. **c** The fitted Baranyi and Roberts model curves per colony [64]; the parameters of the models are provided in Additional file 1: Table S2. Colonies 7, 11, and 12 failed to fit the Baranyi and Roberts model (see text for details)

The model incorporates three bacterial growth stages: lag, exponential, and stationary (Fig. 11a). Let us call *y* the number of cells (in logarithmic scale) and *t* the time in hours. Then $log_{10} N_{0}$ is the starting number of cells, and $log_{10} N_{max}$ the maximum number of cells in the stationary phase (both in logarithmic (base 10) scale). The parameters of the model are the lag time (*λ*), which is the mean life (in hours) of cells of generation 0, and *μ*_*max*_, which is the maximum specific growth rate in (in 1/hour), i.e., the slope of the curve in the exponential phase.

The model parameters for each colony are estimated using non-linear least-squares [67] and provided in Additional file 1: Table S2. In Fig. 11c, we observe that colonies 7, 11, and 12 failed to fit the Baranyi and Roberts model. Colony 7 has very few cells relative to the rest (Fig. 10a); it just reaches the 5th generation (Fig. 3), which implies that it is still in the exponential growth phase and thus fails to fit the model. On the contrary, colony 2 that successfully fitted the model is transitioning to the stationary phase (Fig. 11b). Colonies 11 and 12 failed to fit the model since they did not grow and remained single-cells (notice that in the bar plots of Fig. 10a, and the long linear branches in the FLT of Fig. 2).

#### Cell birth length

**Fig. 12:**
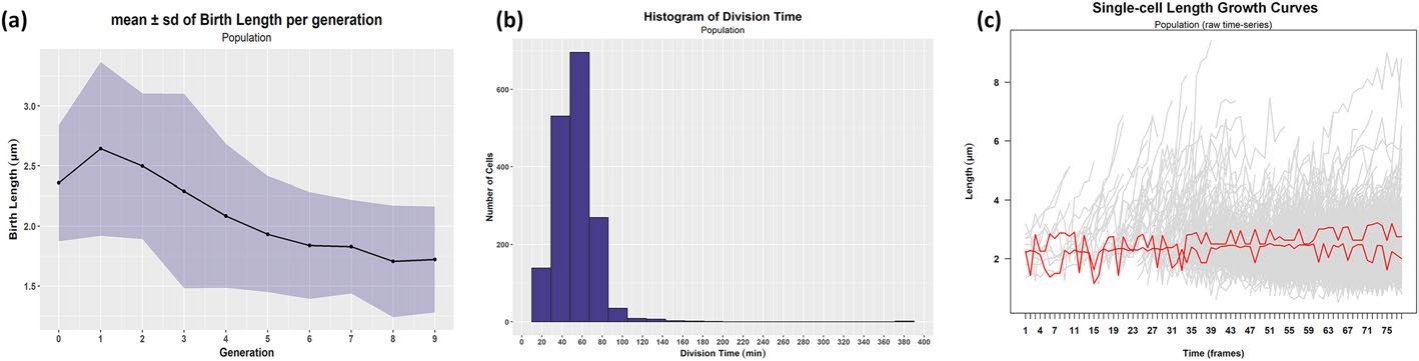
Detection of division time outliers (N = 1695 cells). **a** Variation of the mean birth length of single-cells in successive generations. The shaded area is one standard deviation around the mean (see Additional file 1: Table S3 for details). **b** Histogram (20 bins) of the cell division time. **c** Raw length time-series of cells with division time > 200 min (red lines) compared to the other cells of the population (gray lines)

Cells in the dataset are born with an average length of 1.907 μm (standard deviation of 0.522). However, a closer inspection of the cell birth length variation through successive generations indicates that the mean birth length decreases as the generation index increases (Fig. 12a). Also, there is a trend towards smaller variance. These observations suggest that cells are getting smaller on average, and the population tends to become more homogeneous as time passes.

#### Detection of non-proliferating cells

Entrance of cells into a dormant, non-dividing state is a common adaptive strategy that is largely responsible for several seemingly unrelated puzzling problems in microbiology and the cause of many infectious diseases [5–7]. Therefore, detecting “persisters”, i.e., slowly growing cells that live for many frames, is of great biological interest [6].

Slowly growing cells can be regarded as outliers in terms of their division time. To detect such cells, we created the histogram of the cell division time for the population (Fig. 12b) and selected the cells with division time > 200 min. The threshold was determined by inspecting the histogram. The selected cells’ length time-series visualization reveals two persister-like cells in the dataset that do not grow or divide (Fig. 12c). These outliers correspond to the cells of the non-proliferating colonies 11 and 12.

#### Single-cell attributes variability and modeling

Single-cell analytics offer the capability to characterize the stochasticity of cell (life) attributes and assess their inter- and intra-colony and generation variability. For example, we can inspect the violin plots of the cell division time, cell length at division, cell elongation rate, as well as their corresponding best-fit distributions. The analysis is performed at three different levels of community organization using ViSCAR: population, colony, and generation level. This capability is offered by the plot_viobox_attr function that creates the violin plots and the plot_pdf_attr function that fits a distribution to the data. Since cells of different colonies in this dataset do not represent different strains or species, colony-level analysis is included to illustrate the inherent heterogeneity of a theoretically homogeneous cell population.

The plot_pdf_attr function can be used in the “auto” mode to find the distribution that best fits a cell (life) attribute. The function fits separately the Normal, Gamma, and Lognormal distribution to the corresponding data using maximum likelihood estimation (MLE) [65]. The best-fit distribution is chosen using the Bayesian Information Criterion (BIC) [65], i.e., the best model is the one with the lowest BIC value. To compare the BIC estimates among different distributions, we use ΔBIC. The larger a ΔBIC value of distribution A compared to distribution B, the stronger the evidence that the attribute follows distribution A and not B. ΔBIC values > 10 typically indicate a strong preference for distribution A [65].

**Fig. 13:**
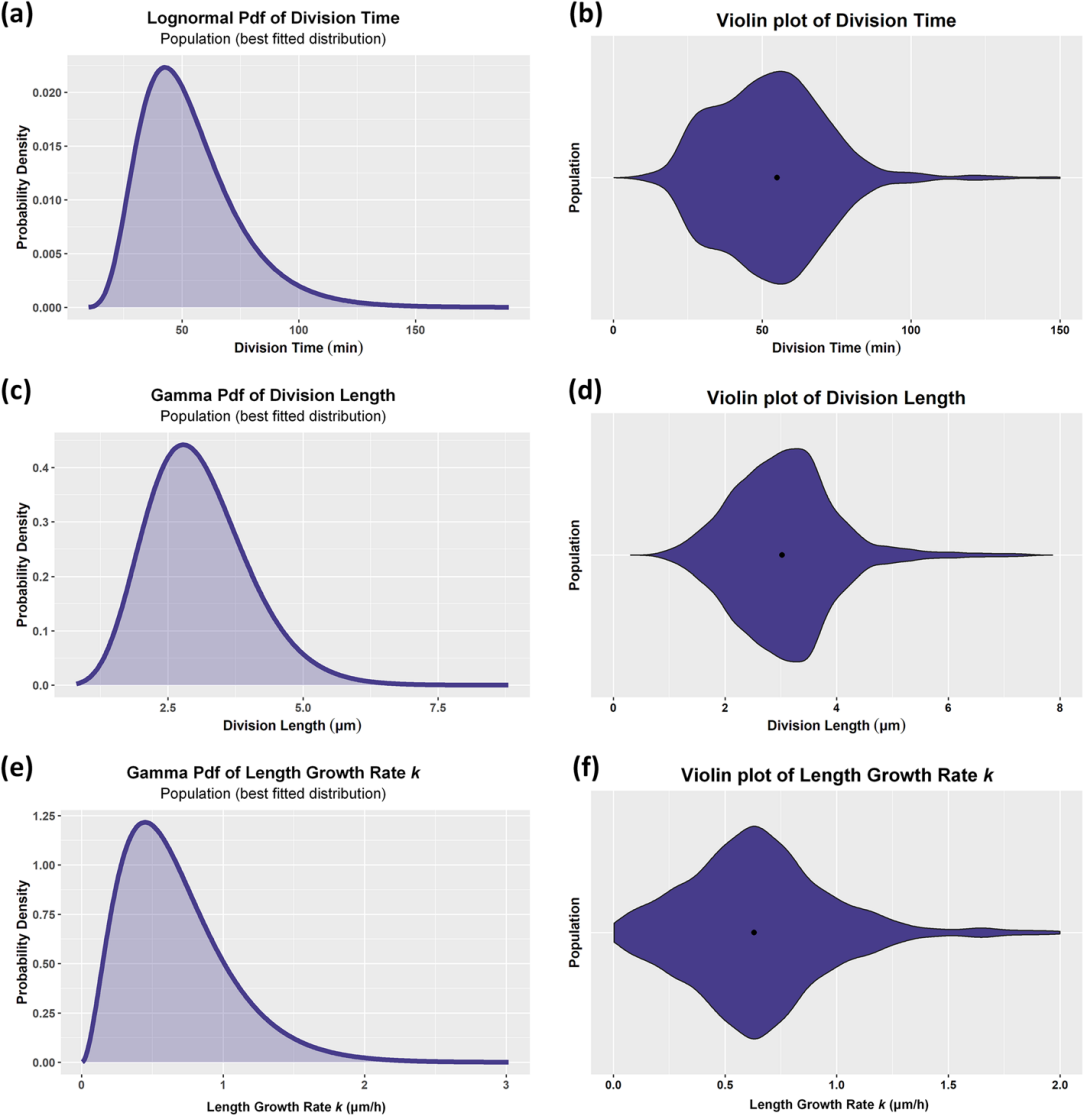
Violin plots and best-fitted distributions on single-cell life attributes for the whole population (N = 1695 cells). Cell division time variability. **a** Best-fit (Lognormal) distribution, **b** Violin plot; multiple modes are apparent in the distribution. Cell length at division variability. **c** Best-fit (Gamma) distribution, **d** violin plot suggests the existence of multiple modes. Cell growth rate *k* variability (N = 1577 cells). **f** Best-fit (Gamma) distribution and **e** violin plot. (See text for details)

Figure 18a presents the best distribution fitting the cell division time data for this complex dataset. This distribution is the Lognormal with parameters μ_log_ = 3.905 and *σ*_*log*_ = 0.388. The fit’s BIC value is 14852, and the ΔBIC is 12 and 567 compared to the Gamma and Normal distributions, respectively. However, in Fig. 13b, we observe that the cell division time distribution is actually a mixture of 2 or more unequally weighted models. This multimodality implies that the division time is distributed differently among different cell subpopulations.

The best-fit distribution for the cell length at division is shown in Fig. 13c. This distribution is the Gamma with parameters *α* = 10.666 and *β* = 3.474. The fit’s BIC value is 4507 and the ΔBIC is 30 and 160 compared to the Lognormal and Normal distribution, respectively. The corresponding violin plot is shown in Fig. 13d.

In Fig. 13e we provide the best distribution fitting the cell exponential elongation rate data, considering only the movie cells that successfully fitted the exponential growth model. Gamma is the best-fit distribution with *α* = 3.026, *β* = 4.520 and BIC value of 1101. The ΔBIC value of the fit is 335 and 429 compared to the Lognormal and Normal distribution, respectively. These large differences indicate that the exponential elongation rate is likely to be Gamma distributed over the population. The corresponding violin plots are shown in Fig. 13d.

The high ΔBIC values of the best-fit distributions compared to the Normal distribution indicate that the three attributes mentioned above deviate significantly from a symmetric distribution. Moreover, the individual cells exhibit substantial variability in terms of their growth kinetics. Multimodality is apparent in the distribution of division time. These observations imply that separate subpopulation analyses are needed to understand the sources of this heterogeneity better. Thus, the multimodality observed in the violin plots of population-level data (Fig. 13) can be attributed to generation- and(or) to colony-level differences for the three examined life attributes.

So, we proceed with analysis per subpopulation to unravel the heterogeneity sources below the population level. We chose the “dominant” best-fit distribution type over the groups (generations or colonies) to facilitate the visual comparison between their distributions, based on Jeffreys’ scale and ΔBIC computation between the model with the lowest BIC value (best fit) and the rest of the models considered. By convention, the Jeffreys’ scale rates ΔBIC > 5 as “strong” and ΔBIC > 10 as “decisive” evidence against the model with the lowest BIC value. So, the most dominant distribution is considered the one which rates with higher frequency ΔBIC ≤ 5. Then, we fitted this distribution to all groups (generations or colonies).

**Fig. 14:**
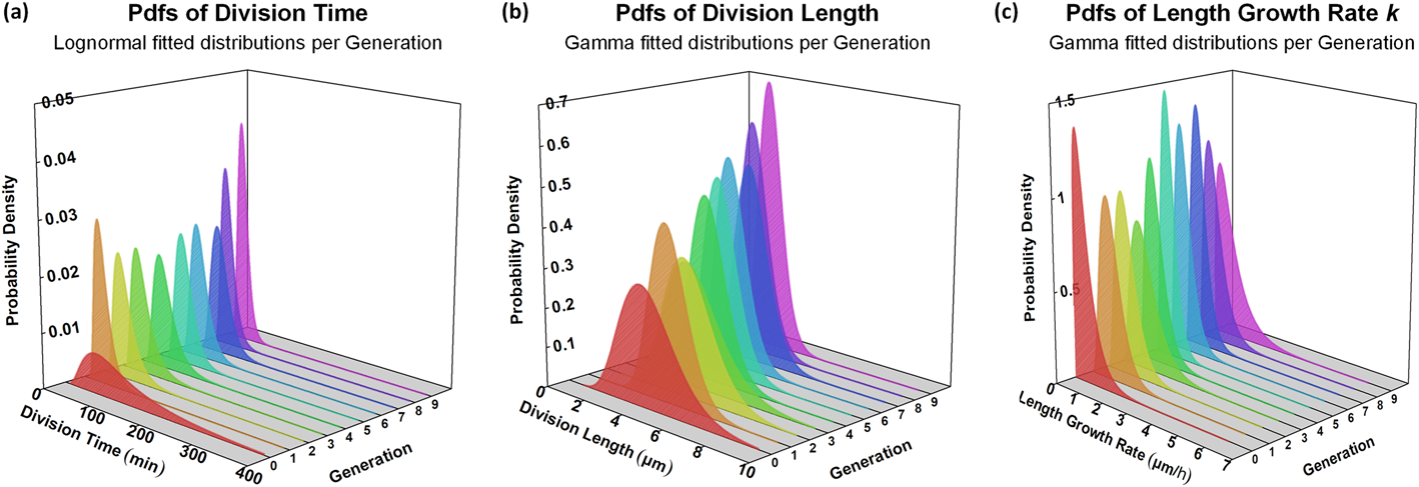
Life attribute distributions per generation. **a** Lognormal distributions fitted on cell division time. **b** Gamma distributions fitted on cell division length. We observe a trend towards a lower mean and variance as the generation index increases. **c** Gamma distributions fitted on cell elongation rate (*k*). We observe a trend towards a specific mean value as the generation index increases. Color represents the different generations in the movie. See Additional file 1: Tables S5, S7, and S9 for the estimated parameters, respectively

The cell division time, cell division length, and growth rate per generation are visualized in Fig. 14, respectively. The results for the cell division time, cell division length, and growth rate per colony are visualized in Additional file 1: Figure S5, respectively. Colonies 7, 11, and 12, which failed to fit the Baranyi and Roberts model, have been excluded from the colony-level statistical analysis.

The parameters and ΔBIC values of each best-fit distribution along with the computed mean and standard deviation per generation (colony) are provided in Additional file 1: Tables S4 (S10), S6 (S12), S8 (S14) for the cell life attributes mentioned above. The mean is equal to *μ* for Normal, α/β for Gamma and ${\text{e}}^{{(\upmu _{\log } +\upsigma _{\log }^{2} /2)}}$ for the Lognormal distribution. Accordingly, the standard deviation is equal to σ for Normal, α/β^2^ for Gamma and $\left( {{\text{e}}^{{\upsigma _{\log }^{2} }} - 1} \right) \cdot {\text{e}}^{{(2 \cdot\upmu _{\log } +\upsigma _{\log }^{2} )}}$ for the Lognormal distribution. The parameters of the finally chosen fitted distributions along with the BIC values of the corresponding model per generation (colony) are summarized in Additional file 1: Tables S5 (S11), S7 (S13), S9 (S15) for the cell life attributes mentioned above, respectively.

The per generation fitted Lognormal distributions of the division time (Fig. 14a) follow a trend to lower mean and standard deviation with some fluctuations as the generation index increases. Moreover, there is a clear trend towards a lower mean and standard deviation for both the cell division length and the growth rate in the per generation distributions (Fig. 14b, c) as the generation index increases. This is also confirmed by the data summarized in Additional file 1: Tables S6 and S8, respectively.

The per colony fitted distributions of the three life attributes can be grouped in the sense that some colonies have very similar distributions for each life attribute (see Additional file 1: Figure S5 and Tables S11, S13, and S15).

**Fig. 15:**
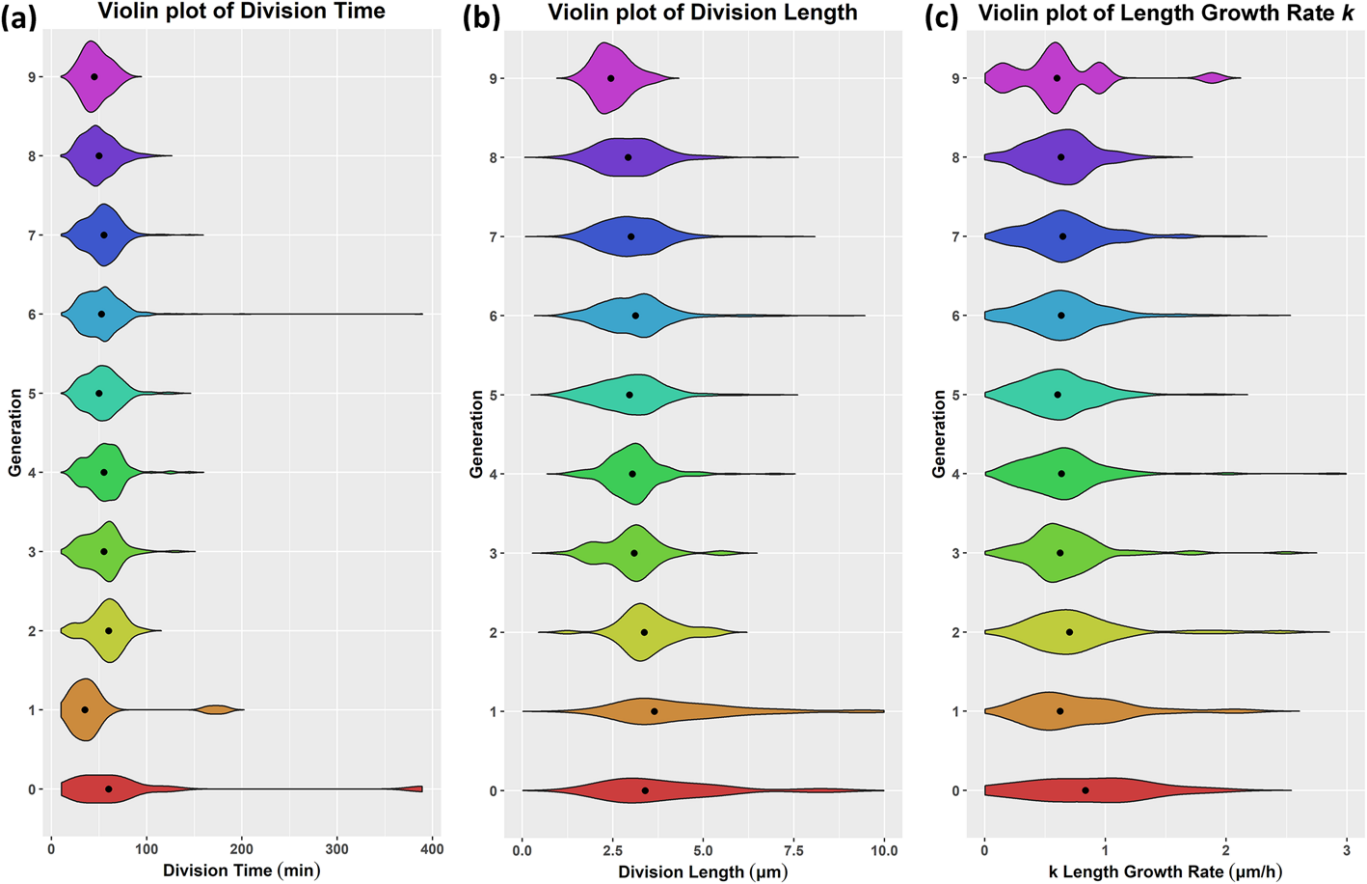
Violin plots per generation. **a** Cell division time. **b** Cell division length. **c** Cell elongation rate (*k*). Color represents the different generations. We observe that multimodality becomes more apparent as the generation index increases

The dominance of the Gamma distribution in most cases hints to multimodality even at the subpopulations level. So, we proceed by examining the violin plots of the subpopulations for the three cell life attributes (division time, division length, elongation rate) per generation (see Fig. 15). The same results per colony are provided in Additional file 1: Figure S6. In Fig. 15a and in Additional file 1: Figure S6a, we observe the cell division time’s multimodal behavior at all levels of community organization. For the cell division length (Fig. 15b and Additional file 1: Figure S6b) and the growth (elongation) rate (Fig. 15c and Additional file 1: Figure S6c), we observe multimodality emerging as generation index is increasing.

These results suggest that as time passes, cells are getting smaller, grow at a lower rate, and at the same time exhibit reduced heterogeneity. This behavior is expected at the population level, and per the Roberts and Baranyi model, the population tends to become more homogeneous as it enters the stationary phase (Fig. 11a). However, only this kind of subpopulation analysis facilitated by ViSCAR enables us to discover high heterogeneity among some colonies and initial generations of the same cell population.

This type of analysis, cutting across community organization levels, demands “deep”, yet accurate, image analysis of large bacterial communities, with thousands of cells, complemented, as here, with suitable analytics to explore the results using a combination of statistical and visualization methods.

### Case study III: Synthetic single-cell movie data set analysis

BaSCA’s capability to analyze single-cell movies (videos) and produce comprehensive representations of the bacterial community organization down to the individual cell instance level, when combined with ViSCAR’s analytics and visualization capabilities pave the road for creating realistic “digital replicas” for studying biological phenomena in silico in the presence of single-cell stochasticity. Their use allows us to transition from the physical world of time-lapse live-cell imaging experiments in the lab to the virtual world of big-data analytics and simulation based on stochasticity-aware individual-based models [31].

We demonstrate here a digital twin prototype of a live-imaging experiment to study the effects of single-cell stochasticity on pathogens’ virulence. To that end, the following pipeline was implemented. We first estimated single-cell growth parameters for *S. Typhimurium* using a single-cell movie (dataset 2) that was image-analyzed using BaSCA and developed mathematical models describing bacterial growth at the single-cell level using ViSCAR. Then, we created a community-level simulation using the CellModeller (see Methods), taking into account the single-cell growth parameters’ stochasticity. In particular, when a mother cell divides, its daughter cells inherit their cell length and elongation rate by drawing samples from the gamma distributions of their generation (see Fig. 15). This action allows us to generate in silico synthetic, yet realistic, bacterial growth datasets of a very large size. We can thus study community behavior at different scales and under different assumptions for the underlying microenvironment. We will demonstrate how VISCAR allowed us to examine the plethora of big-data produced by an in silico proliferating *S. Typhimurium* community and generate new insights, thus closing the systems biology’s loop.

#### Dataset 3 (Synthetic movie)

This very extensive single-cell movie consists of 400 frames acquired with 0.8 min. sampling period. Its main objective is to illustrate the impact of growth parameters’ stochasticity in genetic mechanisms of microbial cells, such as quorum sensing (QS) [76, 77] and the Type Three Secretion System SP-1 (TTSS-1) [77–79] activation. The single-cell movie depicts the simultaneous growth of eight (8) *S. Typhimurium* bacterial populations, each emanating from a single progenitor cell, and was created using an extended version of the CellModeller [51] software tool. The synthetic single-cell movie gets overcrowded, with more than a million cell instances in total. Its 16,964 single-cells have an average length of 2.325 μm and reach the 11th generation. In Additional file 1, we describe thoroughly the scenario of the synthetic movie. In Additional file 4, we provide the synthetic single-cell movie produced from the simulation. Cell surface is colored according to the InvF expression. The dataset needed to reproduce all results of this section is included in https://gitlab.com/ManolakosLab/viscar.

#### Dataset exploration

**Fig. 16:**
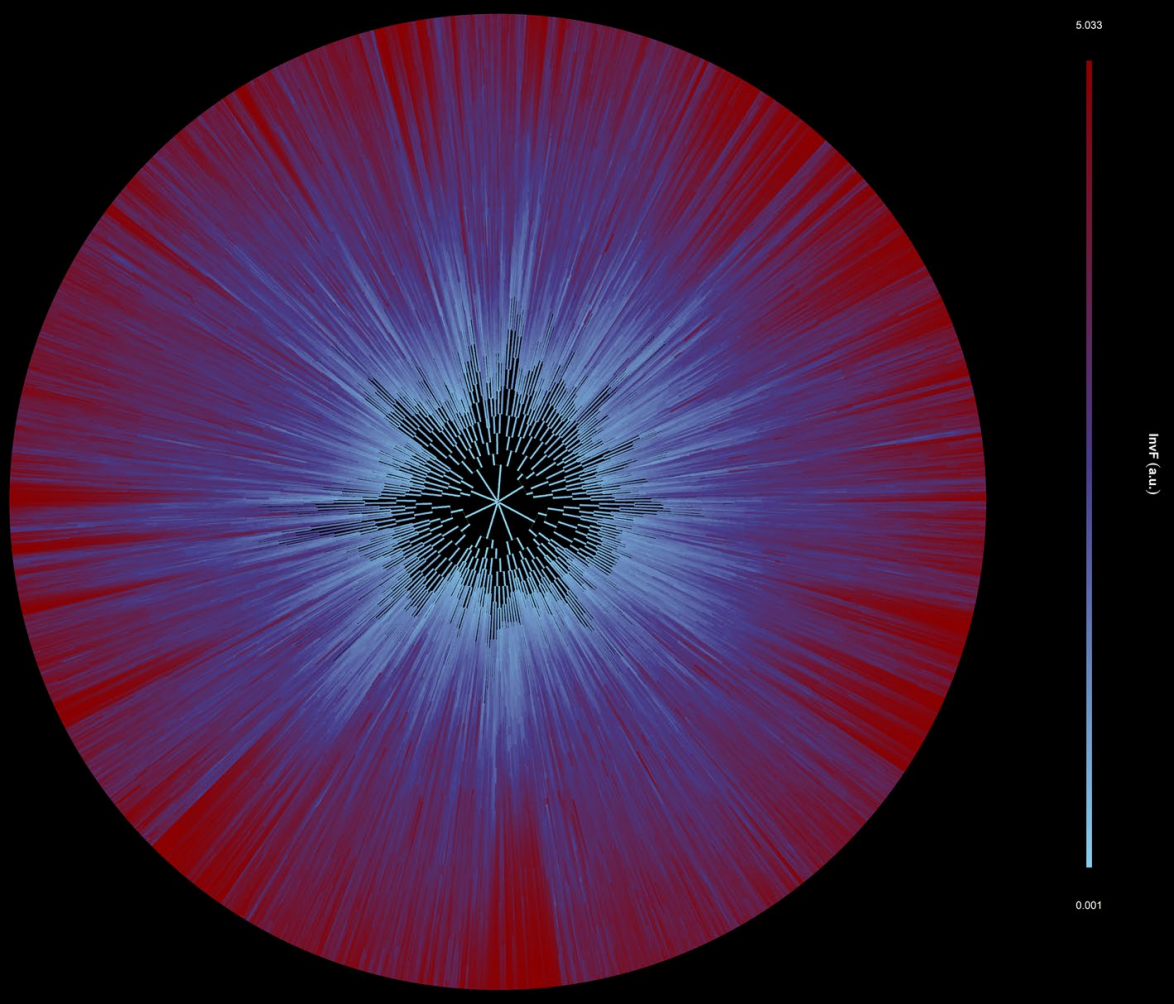
Virulence protein expression mapped on the FLT of the synthetic single-cell movie. Cell instances of the movie are mapped to nodes of the FLT and colored according to InvF (virulence marker) expression levels. Each tree level represents a frame of the single-cell movie (402 levels, 400 frames, and 0.8 min interval between two consecutive frames). The FLT is very dense and deep. The stochasticity in cell growth is reflected in the InvF protein expression patterns among different: (i) trees (colonies), (ii) branches of a tree (cells of a colony), and (iii) levels of the trees (time points, frames)

We load the synthetic single-cell movie dataset 3 (see Methods) and create its FLT representation, see Fig. 16. The distribution of cells to the 11 generations (initial single-cells are counted as the 0th generation) is provided in Additional file 1: Figure S7.

#### Deep forest of trees

**Fig. 17:**
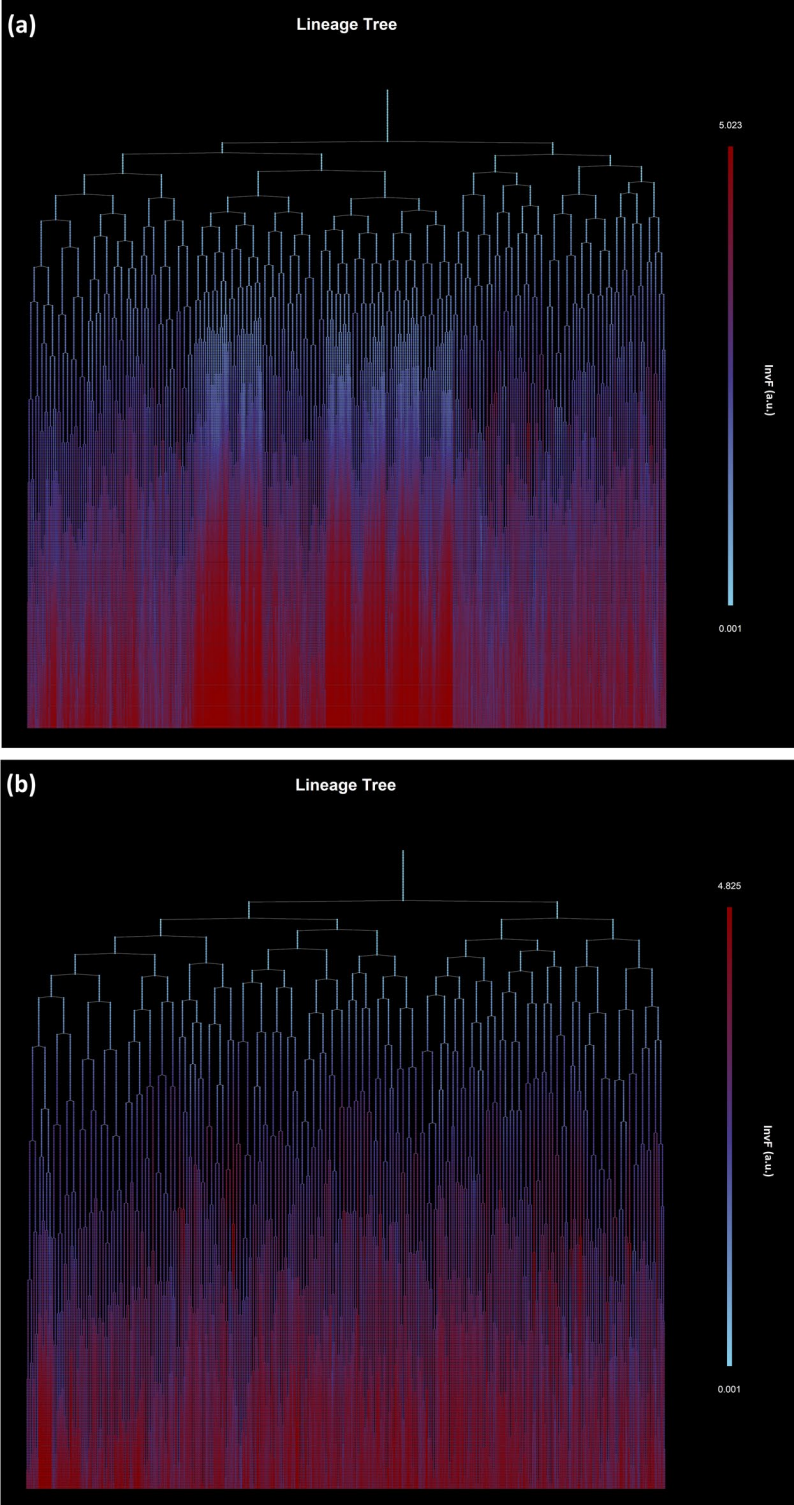
Virulence protein expression patterns in two different cell lineages of the synthetic single-cell movie (cell 3 and cell 7). Cell instances are colored according to a virulence protein’s expression (InvF). Notice the inter- and intra-cell lineage stochasticity in InvF’s expression. **a** In this cell lineage, InvF is highly expressed in some branches but not in others. **b** In this cell lineage, InvF is expressed stochastically, but the variance across the different cell lineages is lower. In both cases, the InvF expression increases with time (more cells switch to a virulent phenotype as time progresses)

As expected for such a complex synthetic movie, the FLT and FDT data structures are very deep and dense due to the dataset having 1,183,630 LT nodes (cell instances). In Fig. 16 we present the FLT, and in Additional file 1: Figure S8 the corresponding FDT having 17,108 nodes (cell lives). A visual inspection of the FLT reveals the heterogeneity immediately in this microbial community. Specifically, in the FLT nodes’ color, we mapped the expression level of the virulence marker, protein InvF. As a cell expresses more highly InvF, it becomes progressively more red (see color bar). The variability in virulence protein’s expression reflects the inter- and intra-subpopulation stochasticity in cell growth (see Additional file 1: Figure S8). In Fig. 17, we zoom into two distinct cell lineages and plot their LTs. We can easily observe that heterogeneity exists among LTs, but also among subtrees of the same LT. In Fig. 17a, we observe that InvF’s expression is significantly higher in some subtrees than in others. On the other hand, in Fig. 17b, the InvF protein is expressed more uniformly across subtrees at lower levels than in Fig. 17a. Due to the stochasticity in cell growth parameters, the cell state heterogeneity leads to different virulence protein expression patterns.

#### Correlation between biophysical and expression cell attributes

ViSCAR enables users to correlate biophysical with expression attributes, e.g., division length with protein concentration at division time at different community organization levels.

**Fig. 18:**
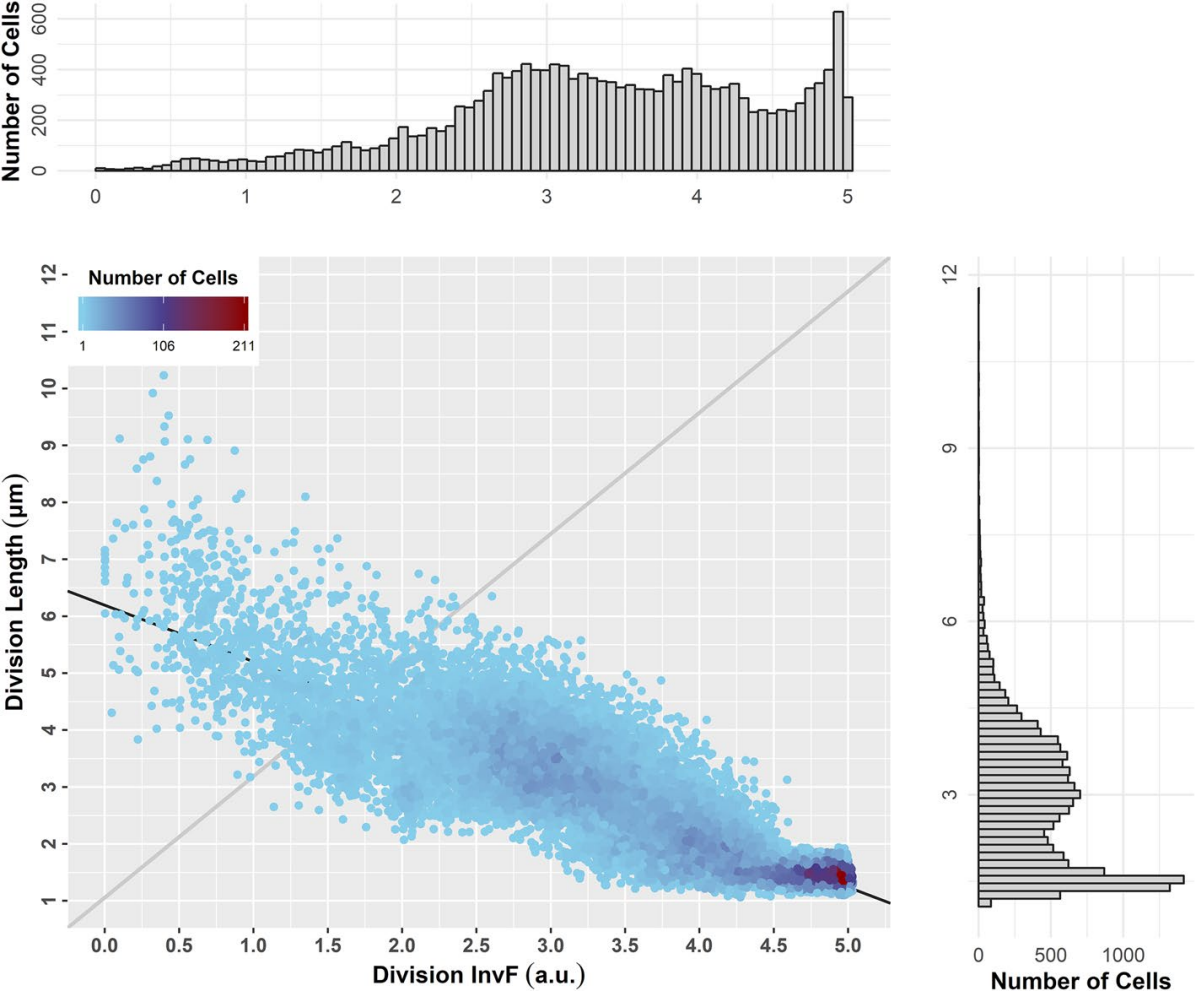
Correlations of biophysical and expression cell life attributes. Scatter plot of cell division length vs. InvF species at division time with regression line (black) *y* = *− *0*.*991* x* + 6*.*196. InvF expression is anti-correlated with division length (*r* = *− *0.849). Color denotes the density of the (*x*, *y*) values (80 bins used for each attribute). Gray lines represent the diagonal of each plot area (bounding box containing all data points). Notice the bimodal population of expressing InvF

For example, in Fig. 18, we present a scatterplot of protein InvF expression (at division) vs. the cell division length for the whole cell population in the movie. We can observe that InvF expression is highly anti-correlated with division length. The multimodality in division length and protein expression is evident in each attribute’s histogram, even at the population level. InvF distribution becomes multimodal as a highly TTSS-1 expressing subpopulation emerges from the whole population.

**Fig. 19:**
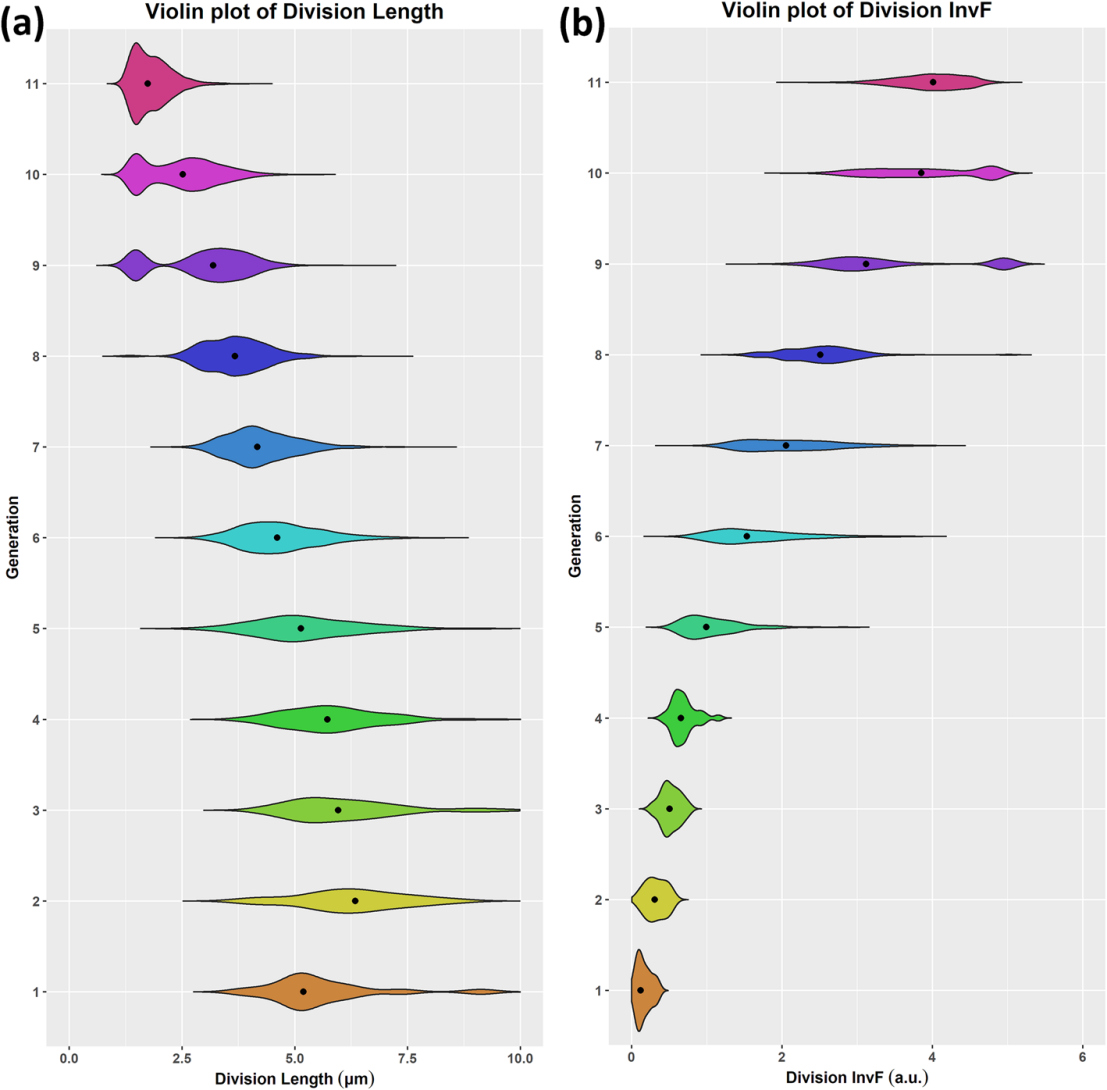
Violin plots of cell life attribute distributions per generation. **a** Cell division length. **b** InvF expression at division time. The color represents the generation index. InvF expression increases on average with the generation index, while division length decreases. InvF expression variance increases with the generation index, while division length variance decreases. Notice that in generations 9 and 10, distributions become clearly bimodal (see text for details)

To explain better the observed multimodality, we must deep dive and examine each attribute’s distribution per cell generation. In Fig. 19, we see the violin plots for division length and InvF expression (at division), respectively, for every cell generation (color). We can observe that as the generation index increases (time passes), the division length decreases on average, as expected in typical microbial growth experiments. On the contrary, the virulence protein expression increases with the generation index. It is evident that the higher the generation index, the higher the variance of protein expression, even though this is not the case for the division length, which becomes smaller and more stable with time. At generations 9 and 10, we can clearly observe the distributions’ bimodality. Bimodality in protein expression is expected because the division length distribution is also bimodal, and protein expression follows the cell growth pattern. In this particular simulation (see Methods), as cells grow, they switch to their virulence phenotype asynchronously, which results in the appearance of a bimodal distribution in cell generations 9 and 10. At the beginning of this movie (first generations), no cells express InvF, while towards the end of the movie (last generations), most of the cells highly express InvF. In the middle part of the movie, the cell population is mixed due to stochasticity in cell growth, which is higher in this section.

### Correlations of life attributes among pairs of cell relatives

**Fig. 20:**
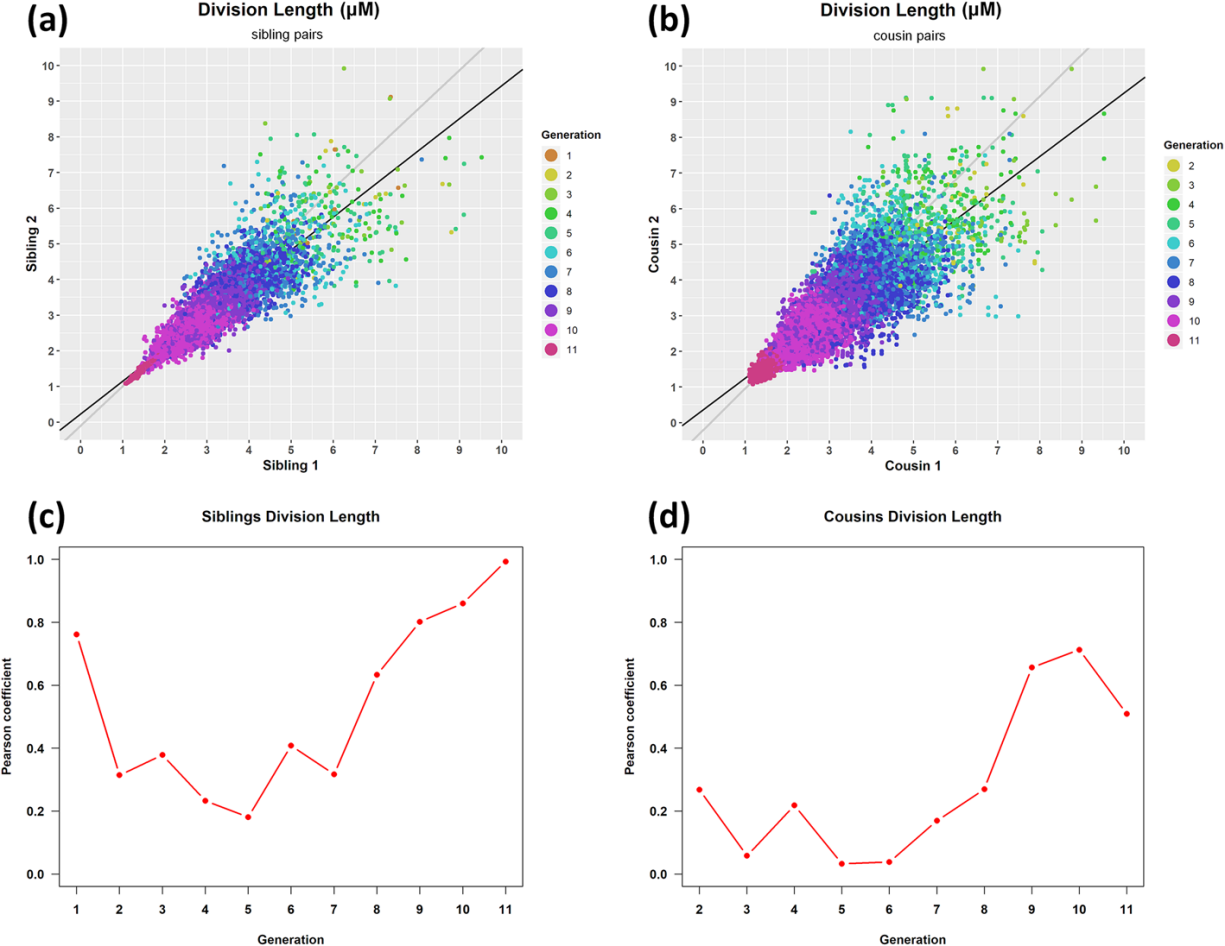
Correlation of division length among pairs of **a** sibling and **b** cousin cells. Color denotes cell generation. Black is the regression (trend) line. Gray is the diagonal of the plot area (bounding box containing all data points). Pearson correlation coefficient of **c** sibling pairs, and **d** cousin pairs per generation. After the 5th generation, correlation increases with the generation index for both relation types. See Additional file 1: Tables S16 and S17 for parameter details

In Fig. 20, we present scatterplots and correlation plots of division length between pairs of related cells of the same generation, namely siblings (having the same mother) or cousins (having the same grandmother) in the single-cell movie. Figure 20a, b show scatterplots for siblings and cousins, respectively; color denotes the generation index. In siblings (cousins) the index starts from 1 (2) because siblings (cousins) exist after the first (second) division of cells, respectively. For siblings, it is evident that as the generation index increases, division length decreases, while the population’s division length becomes more uniform. This pattern applies to cousin pairs too; however, the population is less uniform. In Fig. 20c we present the correlation coefficient of division length between sibling pairs. It is evident that after the 5^th^ generation, the correlation of division length between siblings increases and finally reaches 0.9. The same applies to cousins (see Fig. 20d), even though the correlation coefficient does not reach the same levels. Additional file 1: Tables S16 and S17 provide the correlation coefficients of division length for each generation and the linear regression parameters for the siblings and cousins, respectively. Additional file 1: Tables S18 and S19 provide the correlation coefficients of division time and elongation rate attributes.

In Additional file 1: Tables S16 and S17 we see that siblings are more correlated than cousins in terms of their cell division length since the estimated Pearson’s correlation coefficients are decreasing in that order. The increasing R-squared values assessing the regression line’s goodness-of-fit also confirm this finding. Additionally, the regression line’s slope values are closer to 1 for siblings than for cousins. The same trends are observed for the cell division time (Additional file 1: Tables S18 and S19). These observations suggest that the closer the family relationship between pairs of cells of the same generation, the more “synchronized” they seem to be in their division attributes (time and length).

It seems that siblings become more correlated as the generation index increases in terms of their division time and length. The increase in correlation means that as the bacterial community grows, its local epigenetic “memory” gets better and better. Siblings appear to become more synchronized in their division (time and length) characteristics as the generation index increases [80].

It is worth mentioning that when we applied the same analysis to dataset 2, corresponding to a complex single-cell movie of *S. Typhimurium*, we obtained similar results. Specifically, we observed the same trends in correlations per generation for sibling and cousin pairs in cell division length (Additional file 1: Tables S20 and S21) and cell division time (Additional file 1: Tables S22 and S23).

In https://gitlab.com/ManolakosLab/viscar, we provide the notebooks to reproduce all the results presented in “Results” section.

## Conclusions

We have developed methods and their implementation in the Visualization and Single-Cell Analytics (ViSCAR) R package [81] enabling the transition from the physical world of live-cell imaging experiments (bacterial single-cell movie videos) to the digital world of big data analytics. In that world, ViSCAR can be used to reveal and characterize the stochasticity of single-cell features (morphological, spatial, expression, etc.) allowing researchers to investigate how single-cell heterogeneity may contribute to emerging cellular phenotypes at different scales. These may range from the entire cell population in the single-cell movie, with many interacting colonies and thousands of cells, to flexibly selected cell subpopulations of interest, such as individual micro-colonies, cell generations, cell relatives in consecutive generations, etc. It is worth mentioning, that ViSCAR can be used to perform analytics on communities of other cell types rather than bacterial cells as long as they can be organized on a lineage tree based on some criterion. Except for some ViSCAR capabilities which are specific to bacteria (e.g., fitting Baranyi and Roberts model), the rest of the data analysis and visualization functions are sufficiently general to apply to other cell types.

The presented case studies demonstrated different aspects of the data analyses and how ViSCAR functionalities empower research towards discovering trends and epigenetic effects among relative cells in different cell generations. The R package can be used to identify best models and estimate their parameters characterizing stochastic phenomena (e.g., cell growth, cell division). We can then use the inferred statistical models to draw samples for parameters when running a multi-scale simulation of individual-based models accounting for single-cell stochasticity in the simulation.

There is currently no other R package aiming towards revealing, visualizing, and characterizing single-cell stochasticity in bacterial single-cell movie data sets to the best of our knowledge.

Future work includes an extension to allow the combined analysis of groups of datasets using cloud computing. This will allow creating a “jungle of FLTs” representation of the big data extracted by the image analysis of a collection of single-cell movies corresponding to different live-imaging experiments conducted e.g. for the same species, or under similar environmental/stress conditions.

A unique functionality of ViSCAR is that it can analyze synthetic datasets with many cell generations produced by individual-based modeling and simulation tools, such as the CellModeller [51]. This allows studying in silico behavioral trends of interacting cell populations engineered using different genetic circuits. Such capabilities make ViSCAR appealing also to the synthetic biology community in designing micro-colonies targeting specific biomedical and biotechnological applications.

Finally, by capturing and representing bacterial single-cell movies as Forests of Lineage Trees, in a sense we make the cell moves “eternal.” That means we may archive the corresponding videos since their single-cell multidimensional data representation is fully captured digitally and characterized mathematically. This is a first step towards the generation of “digital twins” for studying a biological phenomenon of interest (e.g., Salmonella persister-cells emergence) using datasets contributed by different labs, which can be shared and easily data-mined by interested researchers over the internet. Such shared digital resources to study cell community dynamics do not exist today because the necessary tools, such as ViSCAR, to create appropriate representations and then data-mine them are currently lacking. We believe that their emergence will accelerate and democratize (crowdsource) science not for the benefit of not only microbial sciences, but also food sciences, systems microbiology, synthetic biology, etc., bringing closer life and computational scientists. We have learned from the ongoing pandemic how important this is for humanity.

## Availability and requirements

- *Project name*: ViSCAR project.
- *Project home page*: https://gitlab.com/ManolakosLab/viscar
- *Operating system(s)*: Platform independent.
- *Programming language*: R
- *Other requirements*: R 3.5.1 or higher.
- *License*: GPL-2.
- *Any restrictions to use by non-academics*: license needed.

## Supplementary Information

**Additional file 1**: Supplementary Information

**Additional file 2**: Synthetic cell instance attribute movie (cell area) of dataset 2

**Additional file 3**: Synthetic cell life attribute movie (cell colony ID) of dataset 2

**Additional file 4**: Synthetic single-cell movie. Cell surface is colored according to InvF expression

## Data Availability

The data sets supporting the results of this article can be downloaded from the ViSCAR’s gitlab repository. The dataset of Case Study I can be also downloaded from SuperSegger website http://mtshasta.phys.washington.edu/website/tutorials.php.

## Abbreviations

BaSCA: Bacterial single cell analytics
ViSCAR: Visualization and single-cell analytics using R
LT: Lineage tree
FLT: Forest of lineage trees
DT: Division tree
FDT: Forest of division trees
PDF: Probability density function
MLE: Maximum likelihood estimation
BIC: Bayesian information criterion
QS: Quorum sensing
TTSS-1: Type three secretion system SP-1
