# Supplementary material for: Analytics and visualization tools to characterize single-cell stochasticity using bacterial single-cell movie cytometry data
Source: BMC Bioinformatics. 2021 Oct 29;22:531. doi: 10.1186/s12859-021-04409-9 (PMC8557071; doi:10.1186/s12859-021-04409-9)
Supplement: Supplementary file 1 — Additional file 1: Supplementary Information [file 12859_2021_4409_MOESM1_ESM.pdf]

Supplementary Information

1. ViSCAR visualization functions

In Table S1, we provide an overview of ViSCAR visualization functions. Each function can provide different levels of analysis. The first two columns (LT and DT) denote the type of input tree, i.e., the type of analyzed attribute(s), cell attributes for an LT, and cell life attributes for a DT. The rest of the columns represent the levels of analysis, spatial (in the sense of community organization**)** and temporal. The ✓ and ✗ symbols indicate whether a visualization capability is available or not. The * symbol indicates that the function produces multiple plot(s). The ~ symbol indicates that a visualization capability can be achieved only indirectly (i.e., not specified by the function's input arguments). Specifically, the user must first specify the subpopulation (using `select_subtree` function) to select a subtree, multiple subtrees, or just not connected nodes of an LT or DT based on multiple selection criteria) and then proceed to create the chosen plot for visual inspection. For example, if the user wants to create a scatter plot for a specific frame then first they must select the cell instants of that frame (using select_subtree) and then proceed with calling the function plot_dot_attr2 with the appropriate arguments.

Table S1. Overview of ViSCAR Functions and their visualization capabilities.

Type	Function	Tree		Spatial		Temporal	
		LT	DT	Population	Colony	Generation	Frame (time)
Scatter plots	`plot_Ncells`	✓	✓	✗	✓	✓	✓
	`plot_dot_attr2`	✓	✓	✓	✓	✓	~
	`plot_dot_attr3`	✓	✓	✓	✓	✓	~
	`plot_dot_time_attr`	✓	✓	✓	~	~	✓
	`plot_dot_attr2_gen2`	✗	✓	✓	~	✓	✗
	`plot_dot_attr_fam`	✗	✓	✓	~	✓	✗
Growth curves	`plot_growth_attr_cell`	✗	✓	✓*	~	~	✓
	`plot_baranyi`	✓	✗	✓	✓*	✗	✓
	`plot_growth_attr`	✗	✓	✓	~	~	✓
	`plot_growth_attr_fit`	✗	✓	✓	✓*	✓*	✗
Statistics	`get_attr_stats`	✓	✓	✓	~	~	~
	`plot_time_attr`	✓	✓	✓	✓*	✓*	✓
	`plot_hist_attr`	✓	✓	✓	✓*	✓*	✗
	`plot_pdf_attr`	✓	✓	✓	✓	✓	✗
	`plot_viobox_attr`	✓	✓	✓	✓	✓	✗

2. Case Study I: Basic workflow and single-cell analytics

In this section we present additional data analysis and visualization operations that users can perform using ViSCAR functions at the cell population and single-cell levels.

In Figure S1, we show the scatter plot of cell length vs. cell area, using color to represent the cell's mean fluorescence intensity. We observe a strong correlation between cell length and cell area, as expected in rod-shaped bacteria. This observation is also confirmed by the very high Pearson correlation coefficient (0.96). In Figure S1b and S1c, we provide the violin plots

of the cell birth length and cell division length, respectively. The observed bimodality is vanished in violin plot of birth length.

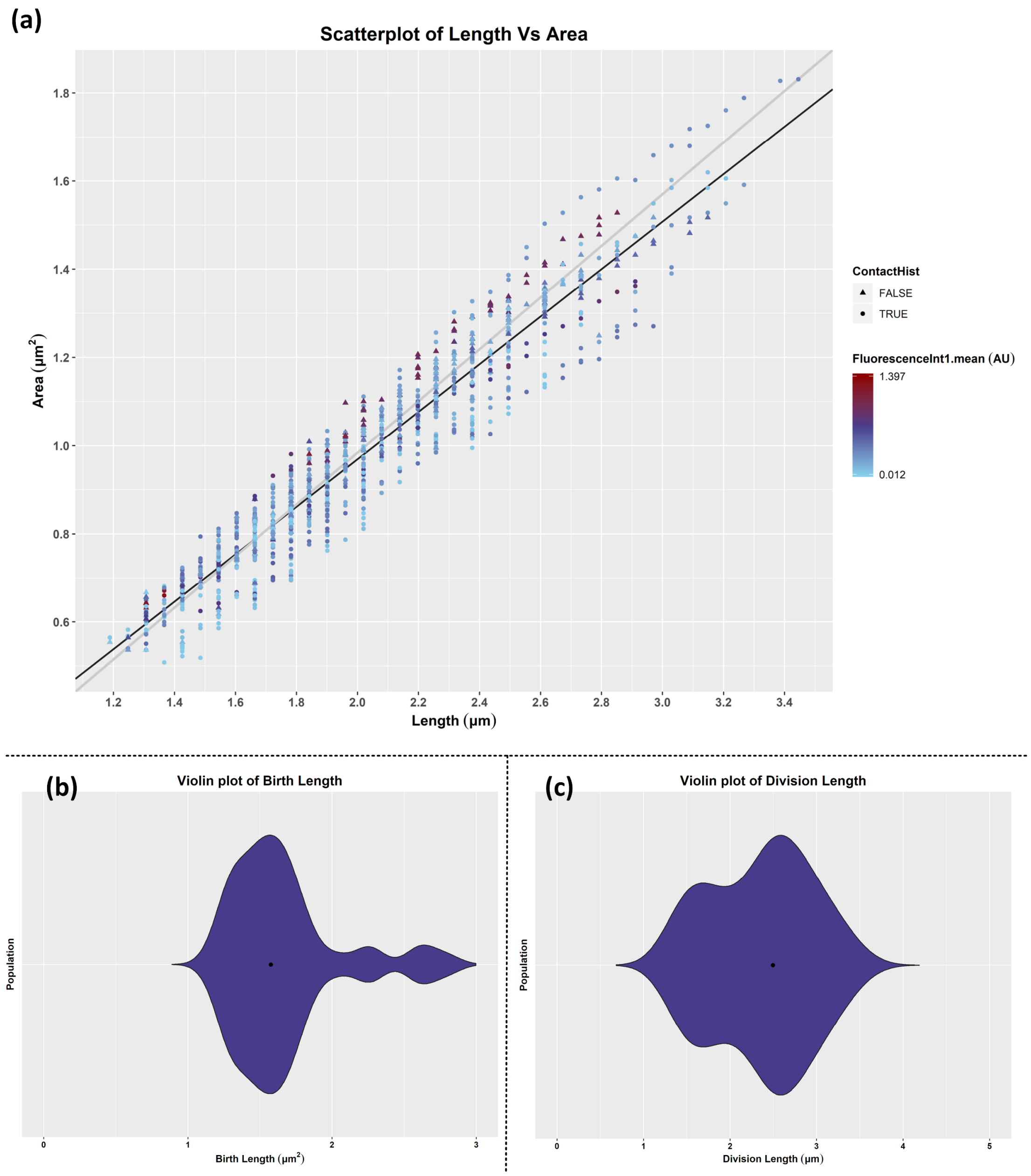

Figure S1. Scatter plot of cell length vs. cell area (dataset 1). Color represents the mean fluorescence intensity of the cell instance (in arbitrary units). The glyph shape indicates whether the cell instance is in contact with other cells or not. The black line is the regression (trend) line. The gray line is the diagonal of the plot area (bounding box containing all data points) (N =

1148). Violin plots of **(b)** cell birth length and **(c)** cell division length (N = 40 cells).

Having computed the cell life attributes, we can detect cells subgroups based on several rules. Given the elongation rate (see Figure tade in main text), we can detect slowly growing cells, such as metabolically inactive cells, see Figure S2. By using the mean and standard deviation method ($< \bar{x} - 2s$) while inspecting the corresponding histogram (Figure S2a) we can detect slowly growing cells, since they are expected to be outliers in terms of their elongation rate. This analysis can reveal persister cells, which are slowly growing cells that live for many frames. However, in this dataset, the only one slowly growing cell detected (**Σφάλμα! Το αρχείο προέλευσης της αναφοράς δεν βρέθηκε.**) cannot be regarded as a persister, as it is tracked for just a few frames because this simple movie ends prematurely. Furthermore, since single-cell length typically increases exponentially [68, 69], cells with abnormal growth curves can be spotted by inspecting the histogram of the RMSE of the exponential models fit to the cell length (Figure S2c). “Abnormal” cells having RMSE > 0.04 were detected; their length time-series (trajectories) were then visualized and compared to the rest of the population (Figure S2d).

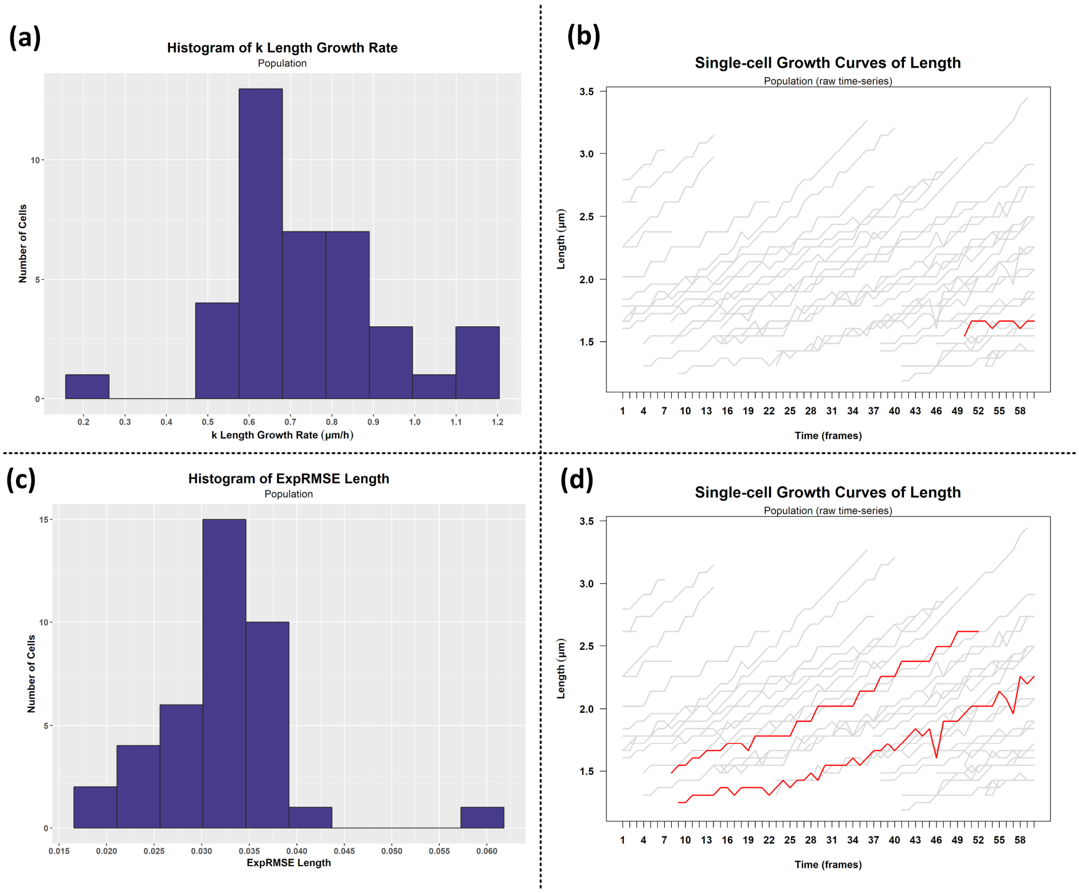

Figure S2. Detection of slowly growing cells (N=41). **(a)** Histogram (10 bins) of the cell exponential elongation rate k. **(b)** Raw data, single-cell length growth curves. The red line depicts the length time-series of slowly growing cells (outliers). Gray curves show the length trajectories of the rest of the cells in the population. Time (x-axis) is the frame index of the single-cell movie. **Detection of cells with irregular growth curves. (c)** Histogram (10 bins) of the RMSE of the exponential fit to the cells' length. **(d)** Length time-series (trajectories) of two cells with exponential fit RMSE >0.04 (red) compared to the rest of the cells in the population (gray). (See text for details).

This kind of processing can reveal interesting patterns of phenotypes, such as stairstep growth behavior (Figure S3a). The large fluctuations observed (Figure

S3b) may be attributed to image analysis imperfections, such as the cell curvature over/underestimation during cell segmentation or cell tracking errors.

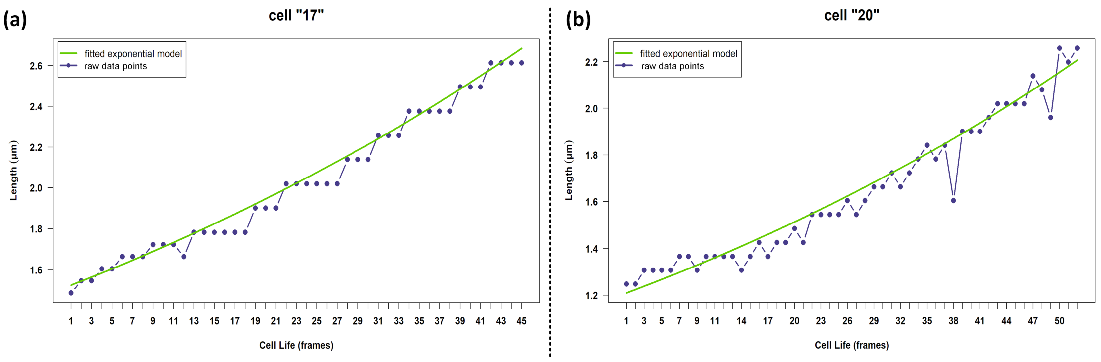

Figure S3. Visualization of the raw cell length (in µm) data (time-series) and the corresponding fitted growth curve for cells with ids 17 in **(a)** and 20 in **(b)** with RMSE exponential fit > 0.04.

The `plot_pdf_attr` function can be used in the "`auto`" mode to find the distribution that best fits a cell (life) attribute. The function fits separately the Normal, Gamma, and Lognormal distribution to the corresponding data using maximum likelihood estimation (MLE) [63]. The best-fit distribution is chosen using the Bayesian Inference Criterion (BIC) [63], i.e., the best model is the one with the lowest BIC value. To compare the BIC estimates among different distributions, we use $\Delta\mathrm{BIC}$. The larger a $\Delta\mathrm{BIC}$ value of distribution A compared to distribution B, the stronger the evidence that the attribute follows distribution A and not B. ΔBIC values >10 typically indicate a strong preference for distribution A [63].

In Figure S4a we present the best-fit distribution for the cell elongation rate k. This distribution is the Normal with $\mu = 0.744$ µm/h and $\sigma = 0.188$, $\mathrm{BIC} = 12.384$, and $\Delta\mathrm{BIC}$ values of 7 and 15 when compared to the Gamma and Lognormal

distributions, respectively. The Gamma and Lognormal (Figure S4b and S4c) distributions were then fitted explicitly to compare them to the best-fit (Normal) distribution. We used a common range for the y-axis of the plots to allow automatic visual comparison. The distribution in Figure S4b is the Gamma with parameters $\alpha = 12.364$, $\beta = 16.626$ and $BIC = 5.306$, and in **Σφάλμα! Το αρχείο προέλευσης της αναφοράς δεν βρέθηκε.**c the Lognormal with parameters $\mu_{log} = 0.337$, $\sigma_{log} = 0.318$ and $BIC = 2.436$.

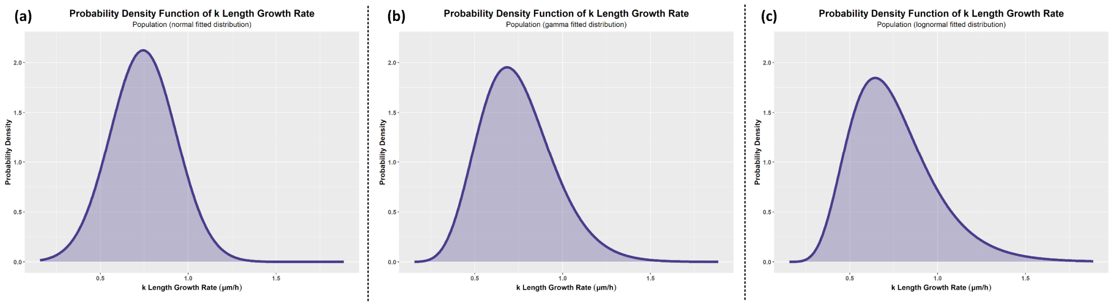

Figure S4. (a) Normal, (b) Gamma, and (c) Lognormal distribution fits the single-cell elongation rate data. Using the BIC criterion, Normal (a) is the best fit distribution for this dataset (see text for details).

It is worth mentioning that the distribution of the cell growth rate k is not normal (symmetrical) in general [71]. Since the difference between the best-fit (Normal) and the Gamma distribution is relatively small ($\Delta BIC = 7$), there is support for that claim in dataset 1.

3. Case study II: Complex single-cell movie analysis results

This section includes supplementary Tables and Figures of the data analysis for Case Study II.

Table S2: Parameters of the fitted Baranyi and Roberts models per colony

Colony	*Lag (h)*	μ_{max} (h^{-1})
1	0.789	1.133
2	1.696	1.422
3	1.006	1.082
4	1.302	1.136
5	0.399	1.162
6	1.325	1.199
8	1.548	1.187
9	0.063	1.018
10	0.758	1.085

Table S3: Estimated mean and standard deviation of the cell birth length (μm) per generation

Generation	No. of cells	Mean	SD
0	12	2.357	0.483
1	20	2.644	0.724
2	40	2.499	0.607
3	80	2.292	0.809
4	152	2.084	0.599
5	290	1.933	0.483
6	492	1.839	0.445
7	383	1.829	0.390
8	206	1.706	0.466
9	20	1.722	0.442

Single-cell attributes variability

For generations:

Table S4: Best-fit distributions of cell division time (min) per generation using BIC

Generation	Distribu-tion	Parameters				ΔBIC Normal	ΔBIC Gamma	ΔBIC log-normal	Mean	SD
**0**	Lognormal	μ_{log}	4.642	σ_{log}	0.666	13	3	-	130	9373

**1**	Lognormal	μ_{log}	3.657	σ_{log}	0.377	7	2	-	42	264
**2**	Lognormal	μ_{log}	3.767	σ_{log}	0.451	28	6	-	48	519
**3**	Gamma	α	4.752	β	0.103	14	-	2	46	446
**4**	Normal	μ	54.013	σ	19.399	-	7	24	54	19
**5**	Lognormal	μ_{log}	4.009	σ_{log}	0.319	90	12	-	58	360
**6**	Gamma	α	8.960	β	0.169	53	-	7	59	312
**7**	Normal	μ	59.178	σ	16.360	-	26	62	59	16
**8**	Lognormal	μ_{log}	3.606	σ_{log}	0.344	53	10	-	39	192
**9**	Lognormal	μ_{log}	3.523	σ_{log}	0.299	4	1	-	35	118

Table S5: Lognormal-fitted distributions of cell division time (min) per generation

Generation	No. of Cells	Parameters		BIC
		μ_{log}	σ_{log}	
**0**	12	4.642	0.666	141
**1**	20	3.657	0.377	170
**2**	40	3.767	0.451	359
**3**	80	3.721	0.482	714
**4**	152	3.912	0.420	1367
**5**	290	4.009	0.319	2497
**6**	492	3.912	0.343	4204
**7**	383	4.037	0.313	3302
**8**	206	3.606	0.344	1641
**9**	20	3.523	0.299	155

Table S6: Best-fit distributions of cell division length (μm) per generation

Generation	Distribu-tion	Parameters				ΔBIC Normal	ΔBIC Gamma	ΔBIC log-norm al	Mean	SD
**0**	Normal	μ	4.287	σ	1.308	-	0	1	4.287	1.308
**1**	Lognormal	μ_{log}	1.444	σ_{log}	0.211	3	1	-	4.331	0.852
**2**	Lognormal	μ_{log}	1.442	σ_{log}	0.266	10	2	-	4.381	1.407
**3**	Lognormal	μ_{log}	1.258	σ_{log}	0.366	16	0	-	3.763	2.032
**4**	Lognormal	μ_{log}	1.230	σ_{log}	0.250	20	2	-	3.531	0.802
**5**	Gamma	α	3.242	β	0.800	0	-	21	3.242	0.800
**6**	Gamma	α	14.159	β	4.902	11	-	15	2.889	0.589
**7**	Normal	μ	3.044	σ	0.771	-	18	48	3.044	0.771

**8**	Lognormal	μ_{log}	0.802	σ_{log}	0.295	42	7	-	2.330	0.493
**9**	Normal	μ	2.264	σ	0.563	-	1	2	2.264	0.563

Table S7: Gamma fitted distributions of cell division length (μm) per generation

Generation	No. of Cells	Parameters		BIC
		α	β	
**0**	12	9.624	2.244	46
**1**	20	22.140	5.109	59
**2**	40	13.612	3.102	133
**3**	80	7.740	2.059	277
**4**	152	16.208	4.590	395
**5**	290	15.529	4.790	708
**6**	492	14.159	4.902	1125
**7**	383	14.162	4.652	918
**8**	206	11.469	4.918	429
**9**	20	14.796	6.536	41

Table S8: Best-fit distributions of cell elongation rate (μm/h) per generation

Generation	Distribu-tion	Parameters				ΔBIC Normal	ΔBIC Gamma	ΔBIC log-normal	Mean	SD
**0**	Normal	μ	0.533	σ	0.251	-	7	14	0.533	0.251
**1**	Normal	μ	0.920	σ	0.318	-	10	19	0.920	0.318
**2**	Normal	μ	0.869	σ	0.355	-	11	24	0.869	0.355
**3**	Lognormal	μ_{log}	-0.216	σ_{log}	0.634	38	1	-	0.985	0.481
**4**	Gamma	α	3.176	β	4.342	47	-	21	0.732	0.168
**5**	Normal	μ	0.623	σ	0.252	-	53	145	0.623	0.252
**6**	Gamma	α	2.995	β	4.713	83	-	82	0.636	0.135
**7**	Gamma	α	3.656	β	5.760	188	-	81	0.635	0.110
**8**	Gamma	α	2.261	β	3.439	36	-	26	0.657	0.191
**9**	Normal	μ	0.735	σ	0.411	-	4	16	0.735	0.411

Table S9: Gamma fitted distributions of cell elongation rate (μm/h) per generation

Generation	No. of Cells	Parameters		BIC
		α	β	
**0**	12	1.488	2.790	12
**1**	20	4.256	4.627	27
**2**	40	3.647	4.199	47
**3**	80	2.764	2.832	117
**4**	152	3.176	4.342	132
**5**	290	4.301	6.909	86
**6**	492	2.995	4.713	284
**7**	383	3.656	5.760	169
**8**	206	2.261	3.439	153
**9**	20	1.439	1.959	27

For Colonies:

Table S10: Best-fit distributions of cell division time (min) per colony

Colony	Distribution	Parameters				ΔBIC Normal	ΔBIC Gamma	ΔBIC lognormal
**1**	Gamma	α	5.978	β	0.111	33	-	7
**2**	Gamma	α	8.130	β	0.143	15	-	2
**3**	Gamma	α	8.943	β	0.162	13	-	1
**4**	Gamma	α	7.999	β	0.149	9	-	2
**5**	Gamma	α	6.135	β	0.120	55	-	8
**6**	Lognormal	μ_{log}	3.940	σ_{log}	0.324	16	0	-
**8**	Normal	μ	51.092	σ	15.985	-	1	8
**9**	Gamma	α	8.588	β	0.157	1	-	22
**10**	Gamma	α	8.213	β	0.165	18	-	6

Table S11: Gamma fitted distributions of cell division time (min) per colony

Colony	No. of Cells	Parameters		BIC
		α	β	
**1**	201	5.978	0.111	1798
**2**	123	8.130	0.143	1083
**3**	117	8.943	0.162	1014
**4**	101	7.999	0.149	882
**5**	349	6.135	0.120	3076
**6**	127	9.822	0.181	1085
**8**	119	9.413	0.184	1008
**9**	281	8.588	0.157	2431
**10**	230	8.213	0.165	1958

Table S12. Best-fit distributions of cell division length (μm) per colony

Colony	Distribution	Parameters				ΔBIC Normal	ΔBIC Gamma	ΔBIC lognormal
**1**	Lognormal	μ_{log}	1.124	σ_{log}	0.325	42	3	-
**2**	Lognormal	μ_{log}	1.133	σ_{log}	0.220	24	5	-
**3**	Normal	μ	2.841	σ	0.800	0	10	20
**4**	Gamma	α	7.898	β	2.644	8	-	3
**5**	Gamma	α	8.993	β	2.814	30	-	6
**6**	Normal	μ	3.023	σ	0.596	0	5	11
**8**	Lognormal	μ_{log}	1.072	σ_{log}	0.296	15	1	-
**9**	Gamma	α	12.355	β	4.078	4	-	13
**10**	Gamma	α	11.260	β	3.834	12	-	7

Table S13. Gamma fitted distributions of cell division length (μm) per colony

Colony	No. of Cells	Parameters		BIC
		α	β	
**1**	201	9.670	2.982	584
**2**	123	20.108	6.313	270
**3**	117	10.896	3.836	299

**4**	101	7.898	2.644	299
**5**	349	8.993	2.814	1020
**6**	127	23.981	7.933	244
**8**	119	11.628	3.809	314
**9**	281	12.355	4.078	710
**10**	230	11.260	3.834	588

Table S14. Best-fit distributions of cell elongation rate (μm/h) per colony

Colony	Distribution	Parameters				ΔBIC Normal	ΔBIC Gamma	ΔBIC lognormal
**1**	Gamma	α	3.525	β	5.064	60	-	8
**2**	Gamma	α	3.506	β	5.139	34	-	12
**3**	Gamma	α	3.403	β	5.681	1	-	26
**4**	Gamma	α	3.454	β	6.198	9	-	16
**5**	Gamma	α	2.675	β	3.567	84	-	94
**6**	Gamma	α	3.612	β	5.875	10	-	19
**8**	Gamma	α	4.191	β	6.643	4	-	14
**9**	Normal	μ	0.677	σ	0.341	-	6	82
**10**	Gamma	α	2.876	β	4.224	141	-	35

Table S15. Gamma fitted distributions of cell elongation rate (μm/h) per colony

Colony	No. of Cells	Parameters		BIC
		α	β	
**1**	201	3.525	5.064	135
**2**	123	3.506	5.139	82
**3**	117	3.403	5.681	47
**4**	101	3.454	6.198	30
**5**	349	2.675	3.567	330
**6**	127	3.612	5.875	57
**8**	119	4.191	6.643	45
**9**	281	3.036	4.486	198
**10**	230	2.876	4.224	174

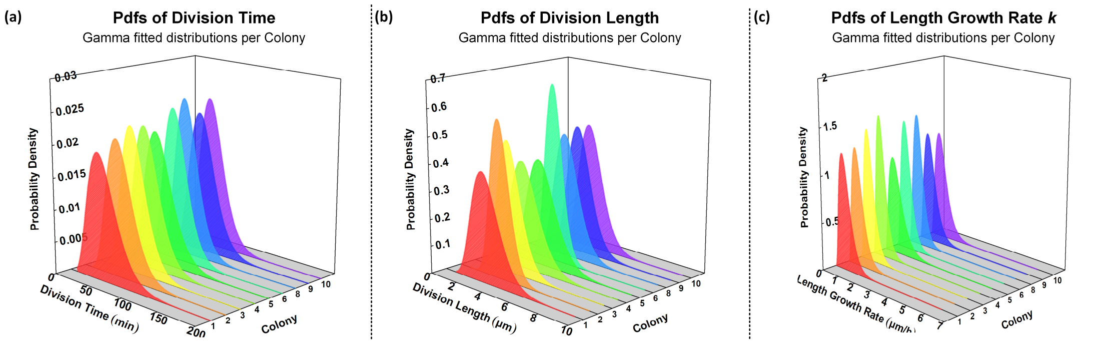

Figure S5. Gamma distributions per colony. (a) Cell division time, **(b)** cell division length, **(c)** cell elongation rate. Color represents the different colonies in the single-cell movie. See Tables 11, 13, and 15 in S1 Text for the estimated parameters, respectively.

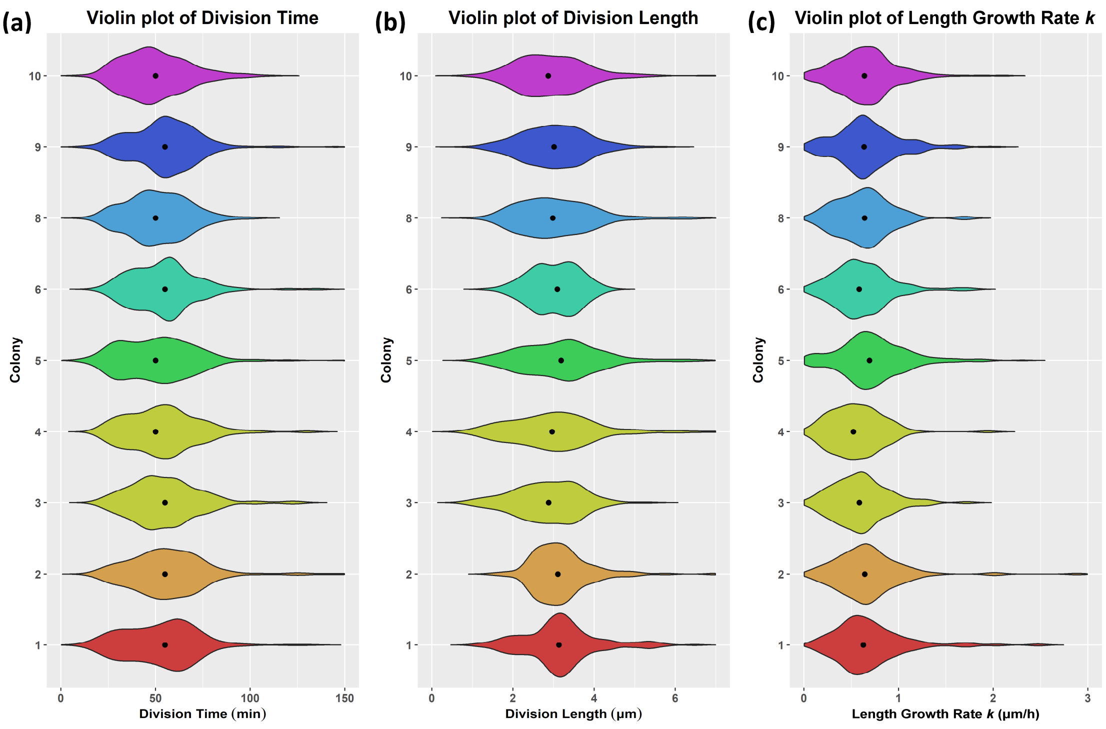

Figure S6. Violin plots per colony. (a) Cell division time, **(b)** cell division length, (c) cell growth (elongation) rate *k*. Color represents the different colonies. We observe that the distributions are multimodal**.**

4. Case study III: Synthetic single-cell movie analysis results

Synthetic single-cell movie

The single-cell movie depicts the simultaneous growth of eight (8) *S. Typhimurium* bacterial populations, each emanating from a single progenitor cell, and was created using an extended version of the CellModeller [50] software tool. By properly extending this tool, we have developed an Individual-Based Model (IBM) and a simulation that mimics QS molecules' (AI_2) [76] diffusion in the microenvironment and shows single-cells that either swim or grow and divide, sensing the QS molecules. As a cell grows, it implements a genetic circuit (described as a system of differential equations) driven by QS that controls the interplay of the TTSS-1 with the virulence pathway [78]. This may eventually lead to a phenotypic switching of a cell from non-virulent to virulent (The development of the IBM is beyond the scope of this work). The key proteins participating in the cell's genetic network are the Lsr operon (LsrOP), LsrR and InvF, for QS and T3SS-1 respectively. The simulation also includes probes docked on the right side of an agar plate, synthesizing the QS AI_2 signal. In this in silico experiment, we assume that the AI_2-producing probes are uniformly distributed on the surface, controlling AI_2 synthesis, bacteria migration, and eventually phenotypic switching. The motile bacteria (initially lying on the plate's left side, opposite to the probes' area) can express TTSS-1 species but cannot produce AI_2. As the motile bacteria sense AI_2, they diffuse in the environment and swim towards the maximum gradient of AI_2^{out}. As they approach the probes' area, they start growing and, at some point, start expressing their virulent phenotype. To introduce stochasticity in cell growth dynamics, when a cell is born, we sample its elongation rate and division length from Gamma distributions estimated using ViSCAR from the analysis of dataset

2. The cells are colored (blue to red) according to the expression of InvF, the primary regulator of T3SS-1, which plays a virulence orchestrator's role. In the specific scenario, InvF gets highly expressed in some cells after the middle of the movie. The dynamic interplay of TTSS-1⁺ and TTSS-1⁻ individuals in a clonal *S. Typhimurium* population depends on the stochasticity introduced in cell growth and on the LuxS-QS modulation (InvF is repressed by LsrR as presented in [77]). The simulation reveals the bistable dynamics of the underlying cell-level gene regulatory network.

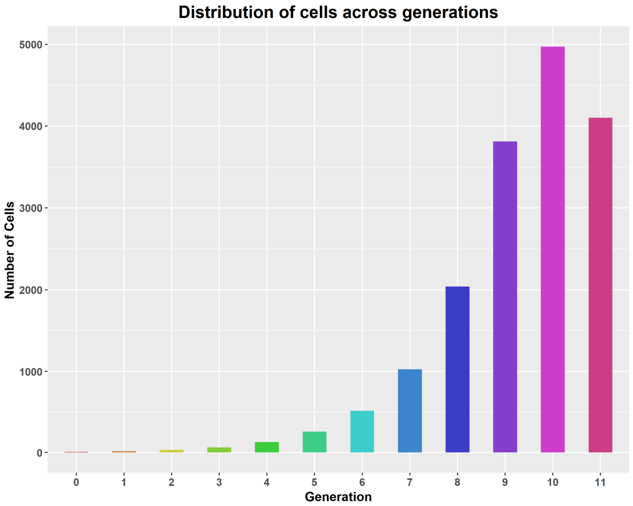

Figure S7. Distribution of cells across generations. Colors represent the generation index. The number of cells per generation increases exponentially.

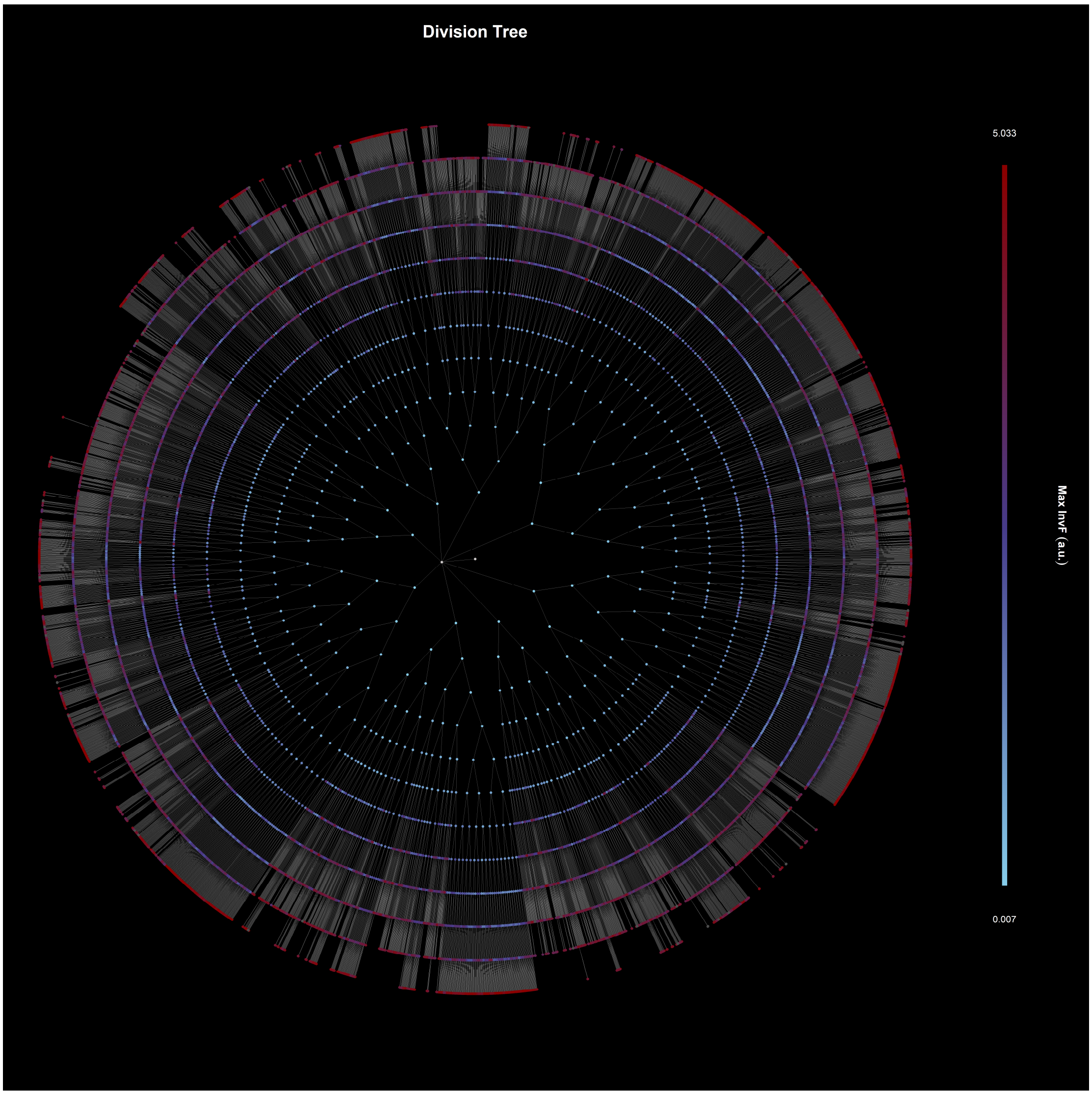

Figure S8. FDT representation and protein expression of the synthetic single-cell movie. Cells of the movie are colored according to the maximum expression of protein InvF (virulence marker). Each level of the tree represents a cell generation (14 levels, 11 generations). The FDT is very deep. Cell growth's parameter variability is reflected in the protein expression heterogeneity across different: i) trees, ii) branches of the same tree, and ii) cell generations.

Table S16. Correlation and regression parameters of siblings per generation for cell division length (dataset 3)

Generation	No. of Pairs	Pearson Corr. Coeff.	Regression parameters		R^2
			Slope (a)	Intercept (b)	
1	8	0.762	1.180	-0.821	0.580
2	16	0.314	0.287	4.060	0.099
3	32	0.379	0.449	3.500	0.144
4	64	0.233	0.178	4.630	0.054
5	128	0.181	0.206	4.390	0.033
6	256	0.408	0.449	2.590	0.167
7	512	0.317	0.318	2.950	0.100
8	1021	0.633	0.609	1.550	0.401
9	1906	0.801	0.818	0.603	0.642
10	2486	0.860	0.847	0.377	0.740
11	2049	0.993	0.992	0.012	0.987

Table S17. Correlation and regression parameters of cousins per generation for cell division length (dataset 3)

Generation	No. of Pairs	Pearson Corr. Coeff.	Regression parameters		R^2
			Slope (a)	Intercept (b)	
2	32	0.268	0.384	3.660	0.072
3	64	0.058	0.043	5.820	0.003
4	128	0.219	0.258	4.620	0.048
5	256	0.032	0.041	5.480	0.001
6	512	0.038	0.043	4.590	0.001
7	1024	0.169	0.167	3.560	0.029
8	2036	0.269	0.303	2.690	0.072
9	3740	0.656	0.725	0.950	0.431
10	4600	0.713	0.765	0.600	0.508
11	3656	0.509	0.539	0.736	0.259

Table S18. Correlation and regression parameters of sibling pairs per generation for cell division time (dataset 3)

Generation	No. of Pairs	Pearson Corr. Coeff.	Regression parameters		R^2
			Slope (a)	Intercept (b)	
1	8	0.394	0.407	9.710	0.156
2	16	0.231	0.362	11.900	0.053
3	32	0.291	0.347	16.100	0.085
4	64	0.552	0.567	9.630	0.305
5	128	0.567	0.643	13.700	0.322
6	256	0.520	0.469	38.100	0.271
7	512	0.548	0.635	37.400	0.300
8	1021	0.705	0.704	31.400	0.497
9	1906	0.787	0.789	23.200	0.619
10	2486	0.878	0.882	9.330	0.771
11	2049	1.000	1.000	2.21E-13	1.000

Table S19. Correlation and regression parameters of cousin pairs per generation for cell division time (dataset 3)

Generation	No. of Pairs	Pearson Corr. Coeff.	Regression parameters		R^2
			Slope (a)	Intercept (b)	
2	32	0.226	0.236	13.500	0.051
3	64	0.201	0.155	19.200	0.040
4	128	0.153	0.194	21.300	0.023
5	256	0.286	0.278	24.800	0.082
6	512	0.316	0.326	49.100	0.100
7	1024	0.343	0.341	69.700	0.118
8	2036	0.478	0.475	60.200	0.228
9	3740	0.602	0.579	41.400	0.363
10	4600	0.662	0.696	19.000	0.438
11	3656	0.969	1.010	-27.000	0.939

Table S20. Correlation and regression parameters of siblings per generation for cell division length (dataset 2)

Generation	No. of Pairs	Pearson Corr. Coeff.	Regression parameters		R^2
			Slope (a)	Intercept (b)	
1	10	0.156	0.138	3.790	0.024
2	20	0.152	0.170	3.757	0.023
3	40	0.218	0.167	3.060	0.048
4	76	0.133	0.175	2.920	0.018
5	145	0.231	0.280	2.332	0.053
6	246	0.494	0.480	1.573	0.244
7	191	0.505	0.449	1.724	0.255
8	102	0.476	0.467	1.204	0.227
9	10	0.659	0.635	0.978	0.434

Table S21. Correlation and regression parameters of cousins per generation for cell division length (dataset 2)

Generation	No. of Pairs	Pearson Corr. Coeff.	Regression parameters		R^2
			Slope (a)	Intercept (b)	
2	40	-0.069	-0.070	4.680	0.005
3	80	0.331	0.628	1.400	0.110
4	152	-0.040	-0.042	3.710	0.002
5	284	0.188	0.174	2.740	0.035
6	464	0.430	0.490	1.450	0.185
7	354	0.280	0.331	2.070	0.078
8	148	0.430	0.496	1.220	0.185
9	12	0.140	0.181	1.320	0.019

Table S22. Correlation and regression parameters of siblings per generation for cell division time (dataset 2)

Generation	No. of Pairs	Pearson Corr. Coeff.	Regression parameters		R^2
			Slope (a)	Intercept (b)	
1	10	0.119	0.171	38.400	0.014
2	20	-0.216	-0.424	72.099	0.046
3	40	0.521	0.405	29.435	0.271
4	76	0.530	0.462	28.861	0.281
5	145	0.226	0.210	45.310	0.051
6	246	0.513	0.533	23.640	0.264
7	191	0.633	0.573	24.615	0.400
8	102	0.721	0.750	9.304	0.520
9	10	0.786	1.228	-3.630	0.618

Table S23. Correlation and regression parameters of cousins per generation for cell division time (dataset 2)

Generation	No. of Pairs	Pearson Corr. Coeff.	Regression parameters		R^2
			Slope (a)	Intercept (b)	
2	40	0.161	0.238	35.700	0.026
3	80	-0.011	-0.019	48.600	0.000
4	152	0.445	0.479	25.800	0.198
5	284	0.123	0.087	50.900	0.015
6	464	0.379	0.413	28.000	0.143
7	354	0.397	0.377	35.500	0.158
8	148	0.523	0.517	16.100	0.274
9	12	0.316	0.200	20.000	0.100
